# Supplementary material for: Structural Diversity of Mercury(II) Halide Complexes Containing Bis-pyridyl-bis-amide with Bulky and Angular Backbones: Ligand Effect and Metal Sensing
Source: Int J Mol Sci. 2022 Jul 16;23(14):7861. doi: 10.3390/ijms23147861 (PMC9323401; doi:10.3390/ijms23147861)

checkCIF/PLATON report

Structure factors have been supplied for datablock(s) 1, 2, 3, 4, 5, 6, 7, 8, 9

THIS REPORT IS FOR GUIDANCE ONLY. IF USED AS PART OF A REVIEW PROCEDURE FOR PUBLICATION, IT SHOULD NOT REPLACE THE EXPERTISE OF AN EXPERIENCED CRYSTALLOGRAPHIC REFEREE.

No syntax errors found. CIF dictionary Interpreting this report

Datablock: 1

```
Bond precision:   C-C = 0.0021 A          Wavelength=0.71073

Cell:           a=8.7814(14)       b=10.5498(16)     c=10.9631(16)
                alpha=85.121(4)    beta=74.166(4)    gamma=81.889(4)
Temperature:    100 K

                 Calculated                         Reported
Volume           966.2(3)                           966.2(3)
Space group      P -1                               P -1
Hall group       -P 1                               -P 1
Moiety formula   C44 H32 Cl2 Hg N8 O8               ?
Sum formula      C44 H32 Cl2 Hg N8 O8               C44 H32 Cl2 Hg N8 O8
Mr               1072.27                            1072.26
Dx,g cm-3        1.843                              1.843
Z                1                                  1
Mu (mm-1)        4.192                              4.192
F000             530.0                              530.0
F000'            528.15
h,k,lmax         11,14,14                           11,14,14
Nref             4826                               4816
Tmin,Tmax        0.380,0.432                        0.508,0.746
Tmin'            0.180

Correction method= # Reported T Limits: Tmin=0.508 Tmax=0.746
AbsCorr = MULTI_SCAN

Data completeness= 0.998            Theta(max)= 28.364

R(reflections)= 0.0142( 4815)                          wR2(reflections)=
                                                       0.0368( 4816)
S = 1.088                    Npar= 286
```

The following ALERTS were generated. Each ALERT has the format
test-name_ALERT_alert-type_alert-level.
Click on the hyperlinks for more details of the test.

Alert level G

```
PLAT154_ALERT_1_G The s.u.'s on the Cell Angles are Equal ..(Note)      0.004 Degree
PLAT432_ALERT_2_G Short Inter X...Y Contact  O2       ..C6       .       2.95 Ang.
                                               1-x,1-y,1-z  =      2_666 Check
PLAT432_ALERT_2_G Short Inter X...Y Contact  C6       ..C6       .       3.13 Ang.
                                               1-x,1-y,1-z  =      2_666 Check
PLAT767_ALERT_4_G INS Embedded LIST 6 Instruction Should be LIST 4     Please Check
PLAT793_ALERT_4_G Model has Chirality at C7          (Centro SPGR)          S Verify
PLAT793_ALERT_4_G Model has Chirality at C8          (Centro SPGR)          S Verify
PLAT793_ALERT_4_G Model has Chirality at C9          (Centro SPGR)          R Verify
PLAT793_ALERT_4_G Model has Chirality at C12         (Centro SPGR)          S Verify
PLAT793_ALERT_4_G Model has Chirality at C13         (Centro SPGR)          R Verify
PLAT793_ALERT_4_G Model has Chirality at C14         (Centro SPGR)          R Verify
PLAT883_ALERT_1_G No Info/Value for _atom_sites_solution_primary .     Please Do !
PLAT912_ALERT_4_G Missing # of FCF Reflections Above STh/L=  0.600         10 Note
PLAT941_ALERT_3_G Average HKL Measurement Multiplicity ..........         4.6 Low
PLAT961_ALERT_5_G Dataset Contains no Negative Intensities .......     Please Check
PLAT978_ALERT_2_G Number C-C Bonds with Positive Residual Density.         14 Info
```

0 **ALERT level A** = Most likely a serious problem - resolve or explain
0 **ALERT level B** = A potentially serious problem, consider carefully
0 **ALERT level C** = Check. Ensure it is not caused by an omission or oversight
15 **ALERT level G** = General information/check it is not something unexpected

2 ALERT type 1 CIF construction/syntax error, inconsistent or missing data
3 ALERT type 2 Indicator that the structure model may be wrong or deficient
1 ALERT type 3 Indicator that the structure quality may be low
8 ALERT type 4 Improvement, methodology, query or suggestion
1 ALERT type 5 Informative message, check

Datablock: 2

Bond precision:	C-C = 0.0050 A	Wavelength=0.71073	
Cell:	a=6.80779(8)	b=11.83139(14)	c=14.71857(16)
	alpha=80.4699(6)	beta=85.6414(6)	gamma=81.8897(6)
Temperature:	296 K		

	Calculated	Reported
Volume	1155.78(2)	1155.78(2)
Space group	P -1	P -1
Hall group	-P 1	-P 1
Moiety formula	C22 H16 Br2 Hg N4 O4	?
Sum formula	C22 H16 Br2 Hg N4 O4	C22 H16 Br2 Hg N4 O4
Mr	760.78	760.80
Dx,g cm-3	2.186	2.186
Z	2	2
Mu (mm-1)	10.153	10.153
F000	716.0	716.0
F000'	710.71	
h,k,lmax	9,15,19	9,15,19
Nref	5741	5727
Tmin,Tmax	0.024,0.362	0.320,0.746
Tmin'	0.012	

Correction method= # Reported T Limits: Tmin=0.320 Tmax=0.746
AbsCorr = MULTI_SCAN

Data completeness= 0.998 Theta(max)= 28.297

R(reflections)= 0.0289(5182) wR2(reflections)= 0.0714(5727)

S = 1.070 Npar= 298

The following ALERTS were generated. Each ALERT has the format
test-name_ALERT_alert-type_alert-level.
Click on the hyperlinks for more details of the test.

Alert level C

PLAT972_ALERT_2_C Check Calcd Resid. Dens. 0.89Ang From Hg -2.06 eA-3
PLAT972_ALERT_2_C Check Calcd Resid. Dens. 0.86Ang From Br1 -1.53 eA-3

Alert level G

PLAT004_ALERT_5_G Polymeric Structure Found with Maximum Dimension 1 Info
PLAT154_ALERT_1_G The s.u.'s on the Cell Angles are Equal ..(Note) 0.0006 Degree
PLAT232_ALERT_2_G Hirshfeld Test Diff (M-X) Hg --Br1 . 9.2 s.u.
PLAT232_ALERT_2_G Hirshfeld Test Diff (M-X) Hg --Br2 . 14.5 s.u.
PLAT232_ALERT_2_G Hirshfeld Test Diff (M-X) Hg --N4_a . 6.4 s.u.
PLAT804_ALERT_5_G Number of ARU-Code Packing Problem(s) in PLATON 2 Info
PLAT883_ALERT_1_G No Info/Value for _atom_sites_solution_primary . Please Do !
PLAT910_ALERT_3_G Missing # of FCF Reflection(s) Below Theta(Min). 1 Note
PLAT912_ALERT_4_G Missing # of FCF Reflections Above STh/L= 0.600 13 Note
PLAT941_ALERT_3_G Average HKL Measurement Multiplicity .... 3.7 Low
PLAT978_ALERT_2_G Number C-C Bonds with Positive Residual Density. 3 Info

```
   0 ALERT level A = Most likely a serious problem - resolve or explain
   0 ALERT level B = A potentially serious problem, consider carefully
   2 ALERT level C = Check. Ensure it is not caused by an omission or oversight
  11 ALERT level G = General information/check it is not something unexpected

   2 ALERT type 1 CIF construction/syntax error, inconsistent or missing data
   6 ALERT type 2 Indicator that the structure model may be wrong or deficient
   2 ALERT type 3 Indicator that the structure quality may be low
   1 ALERT type 4 Improvement, methodology, query or suggestion
   2 ALERT type 5 Informative message, check
```

Datablock: 3

```
Bond precision:   C-C = 0.0051 A          Wavelength=0.71073

Cell:          a=9.7275(5)         b=11.5101(8)      c=12.4521(7)
               alpha=106.3362(18)  beta=98.0437(13)  gamma=114.9454(11)
Temperature:   100 K

                Calculated                       Reported
Volume          1158.30(12)                      1158.30(12)
Space group     P -1                             P -1
Hall group      -P 1                             -P 1
Moiety formula  C22 H16 Hg I2 N4 O4              ?
Sum formula     C22 H16 Hg I2 N4 O4              C22 H16 Hg I2 N4 O4
Mr              854.78                           854.78
Dx,g cm-3       2.451                            2.451
Z               2                                2
Mu (mm-1)       9.347                            9.347
F000            788.0                            788.0
F000'           781.86
h,k,lmax        11,14,15                         11,14,15
Nref            4546                             4540
Tmin,Tmax       0.048,0.154                      0.411,0.746
Tmin'           0.017

Correction method= # Reported T Limits: Tmin=0.411 Tmax=0.746
AbsCorr = MULTI_SCAN

Data completeness= 0.999          Theta(max)= 25.998

R(reflections)= 0.0158( 4481)                    wR2(reflections)=
                                                 0.0397( 4540)

S = 1.029                  Npar= 298
```

The following ALERTS were generated. Each ALERT has the format
test-name_ALERT_alert-type_alert-level.
Click on the hyperlinks for more details of the test.

Alert level C

```
PLAT911_ALERT_3_C Missing FCF Refl Between Thmin & STh/L=    0.600          7 Report
```

Alert level G

```
PLAT793_ALERT_4_G Model has Chirality at C7          (Centro SPGR)          R Verify
PLAT793_ALERT_4_G Model has Chirality at C8          (Centro SPGR)          R Verify
PLAT793_ALERT_4_G Model has Chirality at C9          (Centro SPGR)          S Verify
PLAT793_ALERT_4_G Model has Chirality at C12         (Centro SPGR)          R Verify
PLAT793_ALERT_4_G Model has Chirality at C13         (Centro SPGR)          S Verify
PLAT793_ALERT_4_G Model has Chirality at C14         (Centro SPGR)          S Verify
PLAT883_ALERT_1_G No Info/Value for _atom_sites_solution_primary .     Please Do !
PLAT933_ALERT_2_G Number of HKL-OMIT Records in Embedded .res File          7 Note
PLAT965_ALERT_2_G The SHELXL WEIGHT Optimisation has not Converged     Please Check
PLAT967_ALERT_5_G Note: Two-Theta Cutoff Value in Embedded .res ..       52.0 Degree
PLAT978_ALERT_2_G Number C-C Bonds with Positive Residual Density.          7 Info
```

0 **ALERT level A** = Most likely a serious problem - resolve or explain
0 **ALERT level B** = A potentially serious problem, consider carefully
1 **ALERT level C** = Check. Ensure it is not caused by an omission or oversight
11 **ALERT level G** = General information/check it is not something unexpected

1 ALERT type 1 CIF construction/syntax error, inconsistent or missing data
3 ALERT type 2 Indicator that the structure model may be wrong or deficient
1 ALERT type 3 Indicator that the structure quality may be low
6 ALERT type 4 Improvement, methodology, query or suggestion
1 ALERT type 5 Informative message, check

Datablock: 4

Bond precision: C-C = 0.0057 A Wavelength=0.71073

Cell:	a=25.8153(6)	b=7.0635(2)	c=27.1652(7)
	alpha=90	beta=97.5487(12)	gamma=90
Temperature:	296 K		

	Calculated	Reported
Volume	4910.6(2)	4910.5(2)
Space group	C 2/c	C 2/c
Hall group	-C 2yc	-C 2yc
Moiety formula	C48 H40 Cl4 Hg2 N8 O8	?
Sum formula	C48 H40 Cl4 Hg2 N8 O8	C48 H40 Cl4 Hg2 N8 O8
Mr	1399.86	1399.86
Dx,g cm-3	1.893	1.893
Z	4	4
Mu (mm-1)	6.525	6.525
F000	2704.0	2704.0
F000'	2688.84	
h,k,lmax	34,9,36	34,9,36
Nref	6142	6126
Tmin,Tmax	0.462,0.521	0.342,0.746
Tmin'	0.038	

Correction method= # Reported T Limits: Tmin=0.342 Tmax=0.746
AbsCorr = MULTI_SCAN

Data completeness= 0.997 Theta(max)= 28.328

R(reflections)= 0.0380(4934) wR2(reflections)= 0.1001(6126)

S = 1.059 Npar= 316

The following ALERTS were generated. Each ALERT has the format
test-name_ALERT_alert-type_alert-level.
Click on the hyperlinks for more details of the test.

Alert level C

```
PLAT241_ALERT_2_C High   'MainMol' Ueq as Compared to Neighbors of        C22 Check
PLAT242_ALERT_2_C Low    'MainMol' Ueq as Compared to Neighbors of        C20 Check
PLAT906_ALERT_3_C Large K Value in the Analysis of Variance ......      2.200 Check
PLAT911_ALERT_3_C Missing FCF Refl Between Thmin & STh/L=    0.600          9 Report
PLAT972_ALERT_2_C Check Calcd Resid. Dens.  0.89Ang From Hg             -1.64 eA-3
```

Alert level G

```
PLAT232_ALERT_2_G Hirshfeld Test Diff (M-X)  Hg       --Cl1      .        9.8 s.u.
PLAT793_ALERT_4_G Model has Chirality at C8          (Centro SPGR)          R Verify
PLAT793_ALERT_4_G Model has Chirality at C9          (Centro SPGR)          S Verify
PLAT793_ALERT_4_G Model has Chirality at C11         (Centro SPGR)          S Verify
PLAT793_ALERT_4_G Model has Chirality at C14         (Centro SPGR)          R Verify
PLAT793_ALERT_4_G Model has Chirality at C15         (Centro SPGR)          S Verify
PLAT793_ALERT_4_G Model has Chirality at C18         (Centro SPGR)          R Verify
PLAT883_ALERT_1_G No Info/Value for _atom_sites_solution_primary .     Please Do !
PLAT912_ALERT_4_G Missing # of FCF Reflections Above STh/L=  0.600          7 Note
```

```
PLAT933_ALERT_2_G Number of HKL-OMIT Records in Embedded .res File          2 Note
PLAT941_ALERT_3_G Average HKL Measurement Multiplicity ..........         2.8 Low
PLAT978_ALERT_2_G Number C-C Bonds with Positive Residual Density.          3 Info
```

---

0 **ALERT level A** = Most likely a serious problem - resolve or explain
0 **ALERT level B** = A potentially serious problem, consider carefully
5 **ALERT level C** = Check. Ensure it is not caused by an omission or oversight
12 **ALERT level G** = General information/check it is not something unexpected

1 ALERT type 1 CIF construction/syntax error, inconsistent or missing data
6 ALERT type 2 Indicator that the structure model may be wrong or deficient
3 ALERT type 3 Indicator that the structure quality may be low
7 ALERT type 4 Improvement, methodology, query or suggestion
0 ALERT type 5 Informative message, check

Datablock: 5

Bond precision: C-C = 0.0067 A Wavelength=0.71073

Cell:	a=26.208(2)	b=7.1272(7)	c=27.046(3)
	alpha=90	beta=97.866(5)	gamma=90
Temperature:	305 K		

	Calculated	Reported
Volume	5004.4(8)	5004.3(8)
Space group	C 2/c	C 2/c
Hall group	-C 2yc	-C 2yc
Moiety formula	C48 H40 Br4 Hg2 N8 O8	?
Sum formula	C48 H40 Br4 Hg2 N8 O8	C48 H40 Br4 Hg2 N8 O8
Mr	1577.66	1577.70
Dx,g cm-3	2.094	2.094
Z	4	4
Mu (mm-1)	9.383	9.383
F000	2992.0	2992.0
F000’	2970.78	
h,k,lmax	34,9,36	34,9,36
Nref	6320	6265
Tmin,Tmax	0.121,0.391	0.366,0.746
Tmin’	0.052	

Correction method= # Reported T Limits: Tmin=0.366 Tmax=0.746
AbsCorr = MULTI_SCAN

Data completeness= 0.991 Theta(max)= 28.445

R(reflections)= 0.0397(4327) wR2(reflections)= 0.0904(6265)

S = 1.025 Npar= 298

The following ALERTS were generated. Each ALERT has the format
test-name_ALERT_alert-type_alert-level.
Click on the hyperlinks for more details of the test.

Alert level G

```
PLAT083_ALERT_2_G SHELXL Second Parameter in WGHT  Unusually Large       5.07 Why ?
PLAT171_ALERT_4_G The CIF-Embedded .res File Contains EADP Records          1 Report
PLAT793_ALERT_4_G Model has Chirality at C8          (Centro SPGR)          R Verify
PLAT793_ALERT_4_G Model has Chirality at C9          (Centro SPGR)          S Verify
PLAT793_ALERT_4_G Model has Chirality at C15         (Centro SPGR)          S Verify
PLAT793_ALERT_4_G Model has Chirality at C16         (Centro SPGR)          R Verify
PLAT883_ALERT_1_G No Info/Value for _atom_sites_solution_primary .     Please Do !
PLAT912_ALERT_4_G Missing # of FCF Reflections Above STh/L=  0.600         55 Note
PLAT941_ALERT_3_G Average HKL Measurement Multiplicity ...........        3.9 Low
PLAT965_ALERT_2_G The SHELXL WEIGHT Optimisation has not Converged     Please Check
PLAT978_ALERT_2_G Number C-C Bonds with Positive Residual Density.          2 Info
```

---

0 **ALERT level A** = Most likely a serious problem - resolve or explain
0 **ALERT level B** = A potentially serious problem, consider carefully
0 **ALERT level C** = Check. Ensure it is not caused by an omission or oversight
11 **ALERT level G** = General information/check it is not something unexpected

1 ALERT type 1 CIF construction/syntax error, inconsistent or missing data
3 ALERT type 2 Indicator that the structure model may be wrong or deficient
1 ALERT type 3 Indicator that the structure quality may be low
6 ALERT type 4 Improvement, methodology, query or suggestion
0 ALERT type 5 Informative message, check

Datablock: 6

Bond precision: C-C = 0.0053 A Wavelength=0.71073

Cell:	a=26.7552(5)	b=7.2351(1)	c=27.0340(5)
	alpha=90	beta=98.0783(9)	gamma=90
Temperature:	305 K		

	Calculated	Reported
Volume	5181.22(15)	5181.22(15)
Space group	C 2/c	C 2/c
Hall group	-C 2yc	-C 2yc
Moiety formula	C48 H40 Hg2 I4 N8 O8	?
Sum formula	C48 H40 Hg2 I4 N8 O8	C48 H40 Hg2 I4 N8 O8
Mr	1765.66	1765.66
Dx,g cm-3	2.263	2.264
Z	4	4
Mu (mm-1)	8.362	8.362
F000	3280.0	3280.0
F000'	3255.42	
h,k,lmax	35,9,36	35,9,36
Nref	6465	6439
Tmin,Tmax	0.102,0.188	0.383,0.746
Tmin'	0.065	

Correction method= # Reported T Limits: Tmin=0.383 Tmax=0.746
AbsCorr = MULTI_SCAN

Data completeness= 0.996 Theta(max)= 28.339

R(reflections)= 0.0294(5625) wR2(reflections)= 0.0706(6439)

S = 1.033 Npar= 317

The following ALERTS were generated. Each ALERT has the format
test-name_ALERT_alert-type_alert-level.
Click on the hyperlinks for more details of the test.

Alert level C

```
PLAT911_ALERT_3_C Missing FCF Refl Between Thmin & STh/L=    0.600          2 Report
```

Alert level G

```
PLAT083_ALERT_2_G SHELXL Second Parameter in WGHT  Unusually Large      13.85 Why ?
PLAT232_ALERT_2_G Hirshfeld Test Diff (M-X)  Hg       --I2       .        6.0 s.u.
PLAT793_ALERT_4_G Model has Chirality at C8          (Centro SPGR)          R Verify
PLAT793_ALERT_4_G Model has Chirality at C9          (Centro SPGR)          S Verify
PLAT793_ALERT_4_G Model has Chirality at C15         (Centro SPGR)          S Verify
PLAT793_ALERT_4_G Model has Chirality at C16         (Centro SPGR)          R Verify
PLAT883_ALERT_1_G No Info/Value for _atom_sites_solution_primary .     Please Do !
PLAT912_ALERT_4_G Missing # of FCF Reflections Above STh/L=  0.600         24 Note
PLAT941_ALERT_3_G Average HKL Measurement Multiplicity ..........         3.8 Low
PLAT965_ALERT_2_G The SHELXL WEIGHT Optimisation has not Converged     Please Check
PLAT978_ALERT_2_G Number C-C Bonds with Positive Residual Density.          2 Info
```

```
   0 ALERT level A = Most likely a serious problem - resolve or explain
   0 ALERT level B = A potentially serious problem, consider carefully
   1 ALERT level C = Check. Ensure it is not caused by an omission or oversight
  11 ALERT level G = General information/check it is not something unexpected

   1 ALERT type 1 CIF construction/syntax error, inconsistent or missing data
   4 ALERT type 2 Indicator that the structure model may be wrong or deficient
   2 ALERT type 3 Indicator that the structure quality may be low
   5 ALERT type 4 Improvement, methodology, query or suggestion
   0 ALERT type 5 Informative message, check
```

Datablock: 7

```
Bond precision:   C-C = 0.0045 A          Wavelength=0.71073

Cell:             a=18.4293(3)     b=13.6689(2)        c=9.8057(2)
                  alpha=90         beta=90.3635(9)     gamma=90
Temperature:      296 K

                 Calculated                     Reported
Volume           2470.09(7)                     2470.09(7)
Space group      P 21/c                         P 21/c
Hall group       -P 2ybc                        -P 2ybc
Moiety formula   C24 H18 Cl2 Hg N4 O3, H2 O     ?
Sum formula      C24 H20 Cl2 Hg N4 O4           C24 H20 Cl2 Hg N4 O4
Mr               699.93                         699.93
Dx,g cm-3        1.882                          1.882
Z                4                              4
Mu (mm-1)        6.486                          6.486
F000             1352.0                         1352.0
F000'            1344.42
h,k,lmax         24,18,13                       24,18,13
Nref             6169                           6145
Tmin,Tmax        0.164,0.273                    0.286,0.746
Tmin'            0.124

Correction method= # Reported T Limits: Tmin=0.286 Tmax=0.746
AbsCorr = MULTI_SCAN

Data completeness= 0.996           Theta(max)= 28.352

R(reflections)= 0.0285( 5187)                      wR2(reflections)=
                                                   0.0722( 6145)
S = 1.048                 Npar= 324
```

The following ALERTS were generated. Each ALERT has the format
test-name_ALERT_alert-type_alert-level.
Click on the hyperlinks for more details of the test.

Alert level C

```
PLAT414_ALERT_2_C Short Intra D-H..H-X       H3A      ..H17A     .       1.96 Ang.
                                                      x,y,z  =      1_555 Check
```

Alert level G

```
PLAT004_ALERT_5_G Polymeric Structure Found with Maximum Dimension          1 Info
PLAT007_ALERT_5_G Number of Unrefined Donor-H Atoms .............           4 Report
PLAT232_ALERT_2_G Hirshfeld Test Diff (M-X)  Hg       --N1       .        7.0 s.u.
PLAT300_ALERT_4_G Atom Site Occupancy of O4        Constrained at         0.6 Check
PLAT300_ALERT_4_G Atom Site Occupancy of O4'       Constrained at         0.2 Check
PLAT300_ALERT_4_G Atom Site Occupancy of O4"       Constrained at         0.2 Check
PLAT302_ALERT_4_G Anion/Solvent/Minor-Residue Disorder (Resd  2  )       100% Note
PLAT311_ALERT_2_G Isolated Disordered Oxygen Atom (No H's ?) .....        O4' Check
PLAT311_ALERT_2_G Isolated Disordered Oxygen Atom (No H's ?) .....        O4" Check
PLAT883_ALERT_1_G No Info/Value for _atom_sites_solution_primary .     Please Do !
PLAT910_ALERT_3_G Missing # of FCF Reflection(s) Below Theta(Min).          1 Note
PLAT912_ALERT_4_G Missing # of FCF Reflections Above STh/L=  0.600         24 Note
PLAT978_ALERT_2_G Number C-C Bonds with Positive Residual Density.          6 Info
```

0 **ALERT level A** = Most likely a serious problem - resolve or explain
0 **ALERT level B** = A potentially serious problem, consider carefully
1 **ALERT level C** = Check. Ensure it is not caused by an omission or oversight
13 **ALERT level G** = General information/check it is not something unexpected

1 ALERT type 1 CIF construction/syntax error, inconsistent or missing data
5 ALERT type 2 Indicator that the structure model may be wrong or deficient
1 ALERT type 3 Indicator that the structure quality may be low
5 ALERT type 4 Improvement, methodology, query or suggestion
2 ALERT type 5 Informative message, check

Datablock: 8

Bond precision:	C-C = 0.0154 A	Wavelength=0.71073	
Cell:	a=18.2680(16)	b=13.9475(11)	c=9.9861(8)
	alpha=90	beta=91.326(4)	gamma=90
Temperature:	296 K		

	Calculated	Reported
Volume	2543.7(4)	2543.7(4)
Space group	P 21/c	P 21/c
Hall group	-P 2ybc	-P 2ybc
Moiety formula	2(C24 H18 Br2 Hg N4 O3), 0.375(O4), 2(H0.50 O0.25)	?
Sum formula	C48 H37 Br4 Hg2 N8 O8	C24 H20 Br2 Hg N4 O4
Mr	1574.64	788.85
Dx,g cm-3	2.056	2.060
Z	2	4
Mu (mm-1)	9.230	9.230
F000	1490.0	1496.0
F000'	1479.40	
h,k,lmax	22,17,12	22,17,12
Nref	5040	4984
Tmin,Tmax	0.182,0.158	0.437,0.746
Tmin'	0.137	

Correction method= # Reported T Limits: Tmin=0.437 Tmax=0.746
AbsCorr = MULTI_SCAN

Data completeness= 0.989 Theta(max)= 26.054

R(reflections)= 0.0535(3771) wR2(reflections)= 0.1506(4984)

S = 1.044 Npar= 321

The following ALERTS were generated. Each ALERT has the format
test-name_ALERT_alert-type_alert-level.
Click on the hyperlinks for more details of the test.

Alert level C

```
PLAT041_ALERT_1_C Calc. and Reported SumFormula    Strings  Differ     Please Check
PLAT043_ALERT_1_C Calculated and Reported Mol. Weight Differ by ..       3.06 Check
PLAT068_ALERT_1_C Reported F000 Differs from Calcd (or Missing)...     Please Check
PLAT241_ALERT_2_C High   'MainMol' Ueq as Compared to Neighbors of         O2 Check
PLAT242_ALERT_2_C Low    'MainMol' Ueq as Compared to Neighbors of        C13 Check
PLAT334_ALERT_2_C Small Aver. Benzene C-C Dist C7       -C12             1.37 Ang.
PLAT342_ALERT_3_C Low Bond Precision on  C-C Bonds ...............    0.01536 Ang.
PLAT414_ALERT_2_C Short Intra D-H..H-X       H3A       ..H17A     .      1.96 Ang.
                                                        x,y,z  =      1_555 Check
PLAT430_ALERT_2_C Short Inter D...A Contact  O4        ..O4       .      2.85 Ang.
                                                   -1-x,2-y,-z  =      3_475 Check
PLAT906_ALERT_3_C Large K Value in the Analysis of Variance ......      3.209 Check
PLAT911_ALERT_3_C Missing FCF Refl Between Thmin & STh/L=    0.600         12 Report
```

Alert level G

```
FORMU01_ALERT_2_G  There is a discrepancy between the atom counts in the
            _chemical_formula_sum and the formula from the _atom_site* data.
            Atom count from _chemical_formula_sum:C24 H20 Br2 Hg1 N4 O4
            Atom count from the _atom_site data:  C24 H18.5 Br2 Hg1 N4 O4
CELLZ01_ALERT_1_G Difference between formula and atom_site contents detected.
CELLZ01_ALERT_1_G WARNING: H atoms missing from atom site list. Is this intentional?
           From the CIF: _cell_formula_units_Z    4
           From the CIF: _chemical_formula_sum  C24 H20 Br2 Hg N4 O4
           TEST: Compare cell contents of formula and atom_site data

           atom    Z*formula  cif sites diff
           C          96.00     96.00    0.00
           H          80.00     74.00    6.00
           Br          8.00      8.00    0.00
           Hg          4.00      4.00    0.00
           N          16.00     16.00    0.00
           O          16.00     16.00    0.00
PLAT004_ALERT_5_G Polymeric Structure Found with Maximum Dimension          1 Info
PLAT007_ALERT_5_G Number of Unrefined Donor-H Atoms .............          4 Report
PLAT045_ALERT_1_G Calculated and Reported Z Differ by a Factor ...      0.500 Check
PLAT083_ALERT_2_G SHELXL Second Parameter in WGHT  Unusually Large      13.76 Why ?
PLAT300_ALERT_4_G Atom Site Occupancy of O4         Constrained at       0.75 Check
PLAT300_ALERT_4_G Atom Site Occupancy of O4’        Constrained at       0.25 Check
PLAT300_ALERT_4_G Atom Site Occupancy of H4’A       Constrained at       0.25 Check
PLAT300_ALERT_4_G Atom Site Occupancy of H4’B       Constrained at       0.25 Check
PLAT302_ALERT_4_G Anion/Solvent/Minor-Residue Disorder (Resd  2  )       100% Note
PLAT302_ALERT_4_G Anion/Solvent/Minor-Residue Disorder (Resd  3  )       100% Note
PLAT311_ALERT_2_G Isolated Disordered Oxygen Atom (No H’s ?) .....         O4 Check
PLAT415_ALERT_2_G Short Inter D-H..H-X       H15A     ..H4’B     .       0.78 Ang.
                                              x,3/2-y,1/2+z  =      4_576 Check
PLAT415_ALERT_2_G Short Inter D-H..H-X       H17A     ..H4’A     .       2.08 Ang.
                                                -1-x,2-y,-z  =      3_475 Check
PLAT415_ALERT_2_G Short Inter D-H..H-X       H18A     ..H4’A     .       1.47 Ang.
                                                -1-x,2-y,-z  =      3_475 Check
PLAT432_ALERT_2_G Short Inter X...Y Contact  O4’      ..C15      .       2.47 Ang.
                                             x,3/2-y,-1/2+z  =      4_575 Check
PLAT432_ALERT_2_G Short Inter X...Y Contact  O4’      ..C17      .       2.72 Ang.
                                                -1-x,2-y,-z  =      3_475 Check
PLAT432_ALERT_2_G Short Inter X...Y Contact  O4’      ..C18      .       2.76 Ang.
                                                -1-x,2-y,-z  =      3_475 Check
PLAT720_ALERT_4_G Number of Unusual/Non-Standard Labels ..........          2 Note
PLAT870_ALERT_4_G ALERTS Related to Twinning Effects Suppressed ..          ! Info
PLAT883_ALERT_1_G No Info/Value for _atom_sites_solution_primary .     Please Do !
PLAT910_ALERT_3_G Missing # of FCF Reflection(s) Below Theta(Min).          1 Note
PLAT931_ALERT_5_G CIFcalcFCF  Twin Law ( 1 0 0)         Est.d BASF       0.17 Check
PLAT933_ALERT_2_G Number of HKL-OMIT Records in Embedded .res File          1 Note
PLAT941_ALERT_3_G Average HKL Measurement Multiplicity ...........        1.0 Low
PLAT965_ALERT_2_G The SHELXL WEIGHT Optimisation has not Converged     Please Check
```

---

0 **ALERT level A** = Most likely a serious problem - resolve or explain
0 **ALERT level B** = A potentially serious problem, consider carefully
11 **ALERT level C** = Check. Ensure it is not caused by an omission or oversight
28 **ALERT level G** = General information/check it is not something unexpected

7 ALERT type 1 CIF construction/syntax error, inconsistent or missing data

16 ALERT type 2 Indicator that the structure model may be wrong or deficient
5 ALERT type 3 Indicator that the structure quality may be low
8 ALERT type 4 Improvement, methodology, query or suggestion
3 ALERT type 5 Informative message, check

Datablock: 9

Bond precision: C-C = 0.0091 A Wavelength=0.71073

Cell:	a=18.052(3)	b=14.386(3)	c=10.1560(18)
	alpha=90	beta=91.810(7)	gamma=90
Temperature:	296 K		

	Calculated	Reported
Volume	2636.2(8)	2636.3(8)
Space group	P 21/c	P 21/c
Hall group	-P 2ybc	-P 2ybc
Moiety formula	C24 H18 Hg I2 N4 O3, H2 O	?
Sum formula	C24 H20 Hg I2 N4 O4	C24 H20 Hg I2 N4 O4
Mr	882.83	882.83
Dx,g cm-3	2.224	2.224
Z	4	4
Mu (mm-1)	8.217	8.217
F000	1640.0	1640.0
F000’	1627.71	
h,k,lmax	24,19,13	24,19,13
Nref	6583	6571
Tmin,Tmax	0.106,0.193	0.385,0.746
Tmin’	0.068	

Correction method= # Reported T Limits: Tmin=0.385 Tmax=0.746
AbsCorr = MULTI_SCAN

Data completeness= 0.998 Theta(max)= 28.350

R(reflections)= 0.0396(5056) wR2(reflections)= 0.1008(6571)

S = 1.034 Npar= 316

The following ALERTS were generated. Each ALERT has the format
test-name_ALERT_alert-type_alert-level.
Click on the hyperlinks for more details of the test.

Alert level C

```
PLAT241_ALERT_2_C High   'MainMol' Ueq as Compared to Neighbors of        O2 Check
PLAT241_ALERT_2_C High   'MainMol' Ueq as Compared to Neighbors of       C22 Check
PLAT242_ALERT_2_C Low    'MainMol' Ueq as Compared to Neighbors of       C13 Check
PLAT260_ALERT_2_C Large Average Ueq of Residue Including        O4      0.145 Check
PLAT342_ALERT_3_C Low Bond Precision on  C-C Bonds ...............    0.00914 Ang.
PLAT414_ALERT_2_C Short Intra D-H..H-X       H3A      ..H17A     .       1.94 Ang.
                                                  x,y,z  =      1_555 Check
PLAT906_ALERT_3_C Large K Value in the Analysis of Variance ......      2.570 Check
PLAT971_ALERT_2_C Check Calcd Resid. Dens.  0.89Ang From I2              1.68 eA-3
PLAT976_ALERT_2_C Check Calcd Resid. Dens.  0.59Ang From O4      .      -0.45 eA-3
PLAT977_ALERT_2_C Check Negative Difference Density on H4C       .      -0.37 eA-3
PLAT977_ALERT_2_C Check Negative Difference Density on H4D       .      -0.33 eA-3
```

Alert level G

```
PLAT004_ALERT_5_G Polymeric Structure Found with Maximum Dimension          1 Info
PLAT007_ALERT_5_G Number of Unrefined Donor-H Atoms ..............          4 Report
PLAT232_ALERT_2_G Hirshfeld Test Diff (M-X)  Hg       --I2       .        6.3 s.u.
PLAT883_ALERT_1_G No Info/Value for _atom_sites_solution_primary .     Please Do !
PLAT910_ALERT_3_G Missing # of FCF Reflection(s) Below Theta(Min).          1 Note
PLAT912_ALERT_4_G Missing # of FCF Reflections Above STh/L=  0.600         12 Note
PLAT941_ALERT_3_G Average HKL Measurement Multiplicity ...........        4.0 Low
PLAT965_ALERT_2_G The SHELXL WEIGHT Optimisation has not Converged     Please Check
PLAT978_ALERT_2_G Number C-C Bonds with Positive Residual Density.          1 Info
```

---

0 **ALERT level A** = Most likely a serious problem - resolve or explain
0 **ALERT level B** = A potentially serious problem, consider carefully
11 **ALERT level C** = Check. Ensure it is not caused by an omission or oversight
9 **ALERT level G** = General information/check it is not something unexpected

1 ALERT type 1 CIF construction/syntax error, inconsistent or missing data
12 ALERT type 2 Indicator that the structure model may be wrong or deficient
4 ALERT type 3 Indicator that the structure quality may be low
1 ALERT type 4 Improvement, methodology, query or suggestion
2 ALERT type 5 Informative message, check

It is advisable to attempt to resolve as many as possible of the alerts in all categories. Often the minor alerts point to easily fixed oversights, errors and omissions in your CIF or refinement strategy, so attention to these fine details can be worthwhile. In order to resolve some of the more serious problems it may be necessary to carry out additional measurements or structure refinements. However, the purpose of your study may justify the reported deviations and the more serious of these should normally be commented upon in the discussion or experimental section of a paper or in the "special_details" fields of the CIF. checkCIF was carefully designed to identify outliers and unusual parameters, but every test has its limitations and alerts that are not important in a particular case may appear. Conversely, the absence of alerts does not guarantee there are no aspects of the results needing attention. It is up to the individual to critically assess their own results and, if necessary, seek expert advice.

Publication of your CIF in IUCr journals

A basic structural check has been run on your CIF. These basic checks will be run on all CIFs submitted for publication in IUCr journals (*Acta Crystallographica*, *Journal of Applied Crystallography*, *Journal of Synchrotron Radiation*); however, if you intend to submit to *Acta Crystallographica Section C* or *E* or *IUCrData*, you should make sure that full publication checks are run on the final version of your CIF prior to submission.

Publication of your CIF in other journals

Please refer to the *Notes for Authors* of the relevant journal for any special instructions relating to CIF submission.

PLATON version of 19/02/2022; check.def file version of 19/02/2022

Datablock 1 - ellipsoid plot

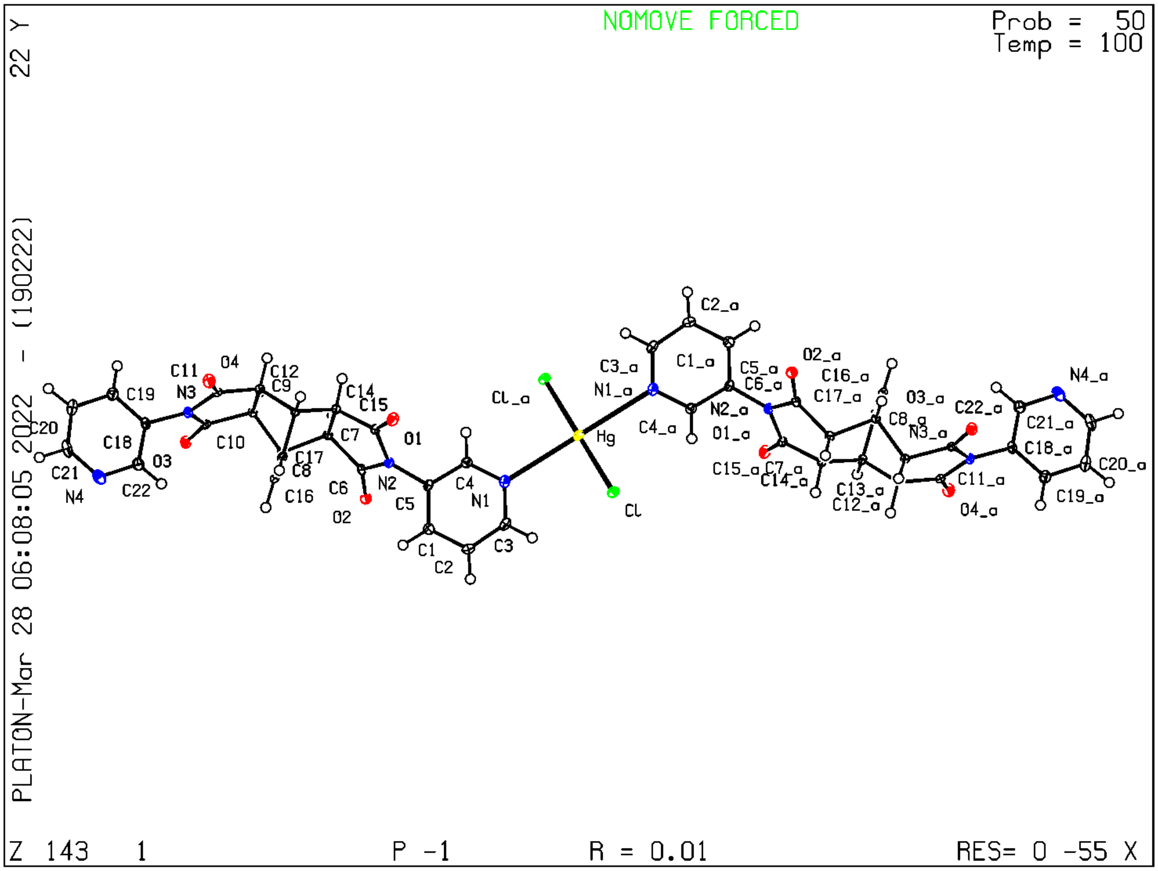

Datablock 2 - ellipsoid plot

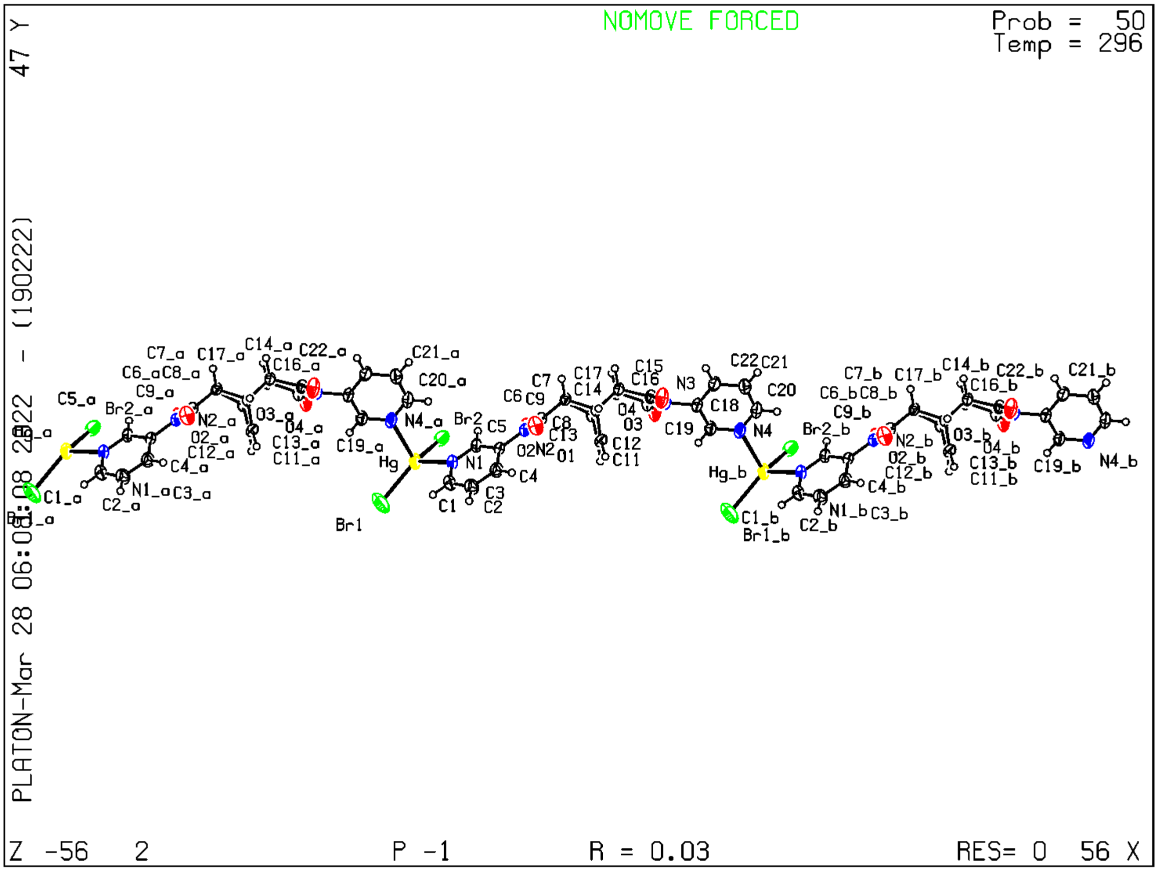

Datablock 3 - ellipsoid plot

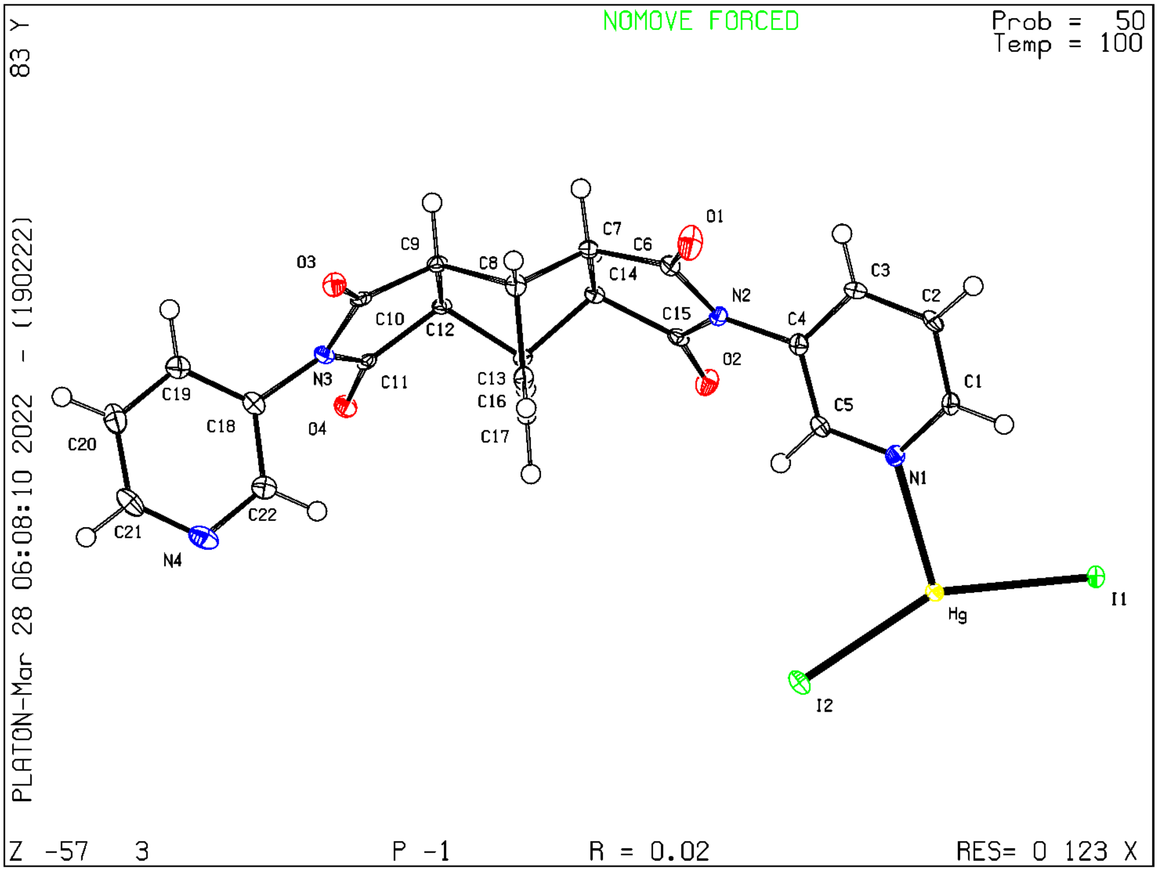

Datablock 4 - ellipsoid plot

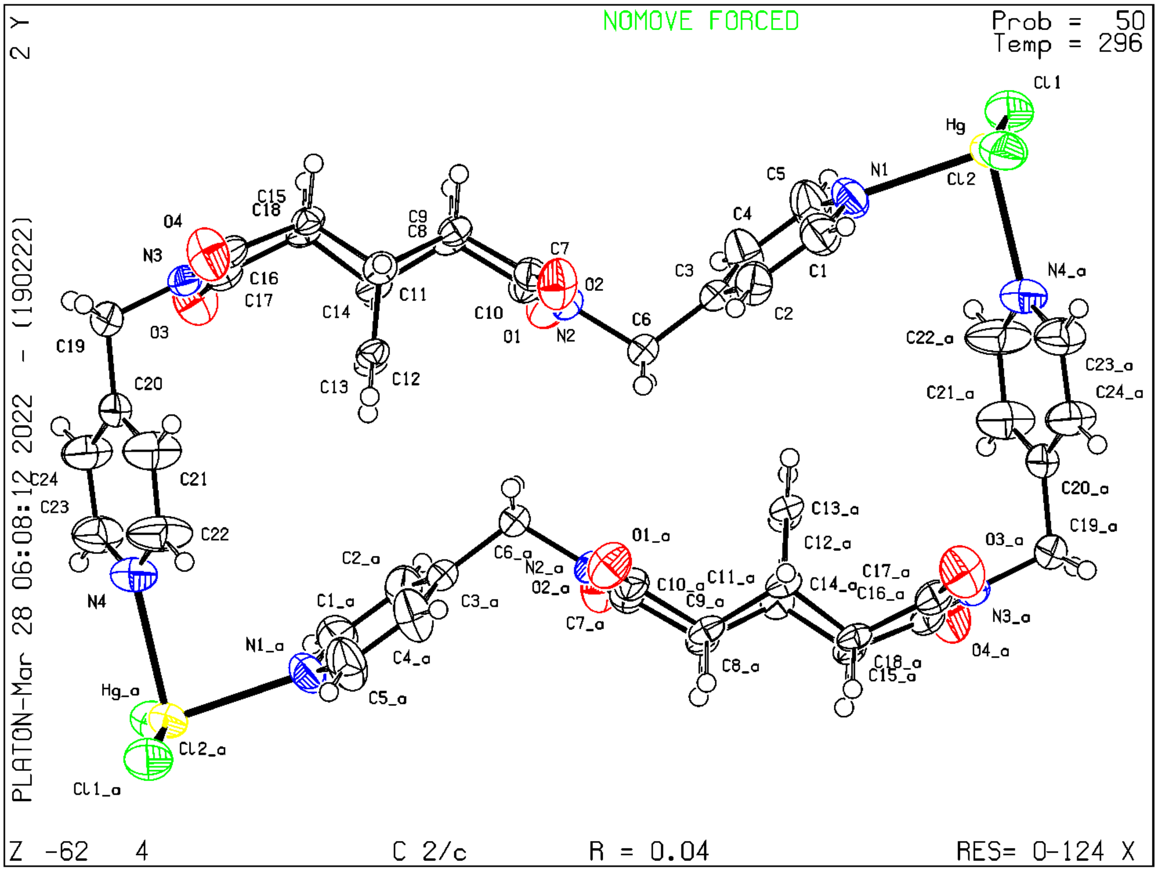

Datablock 5 - ellipsoid plot

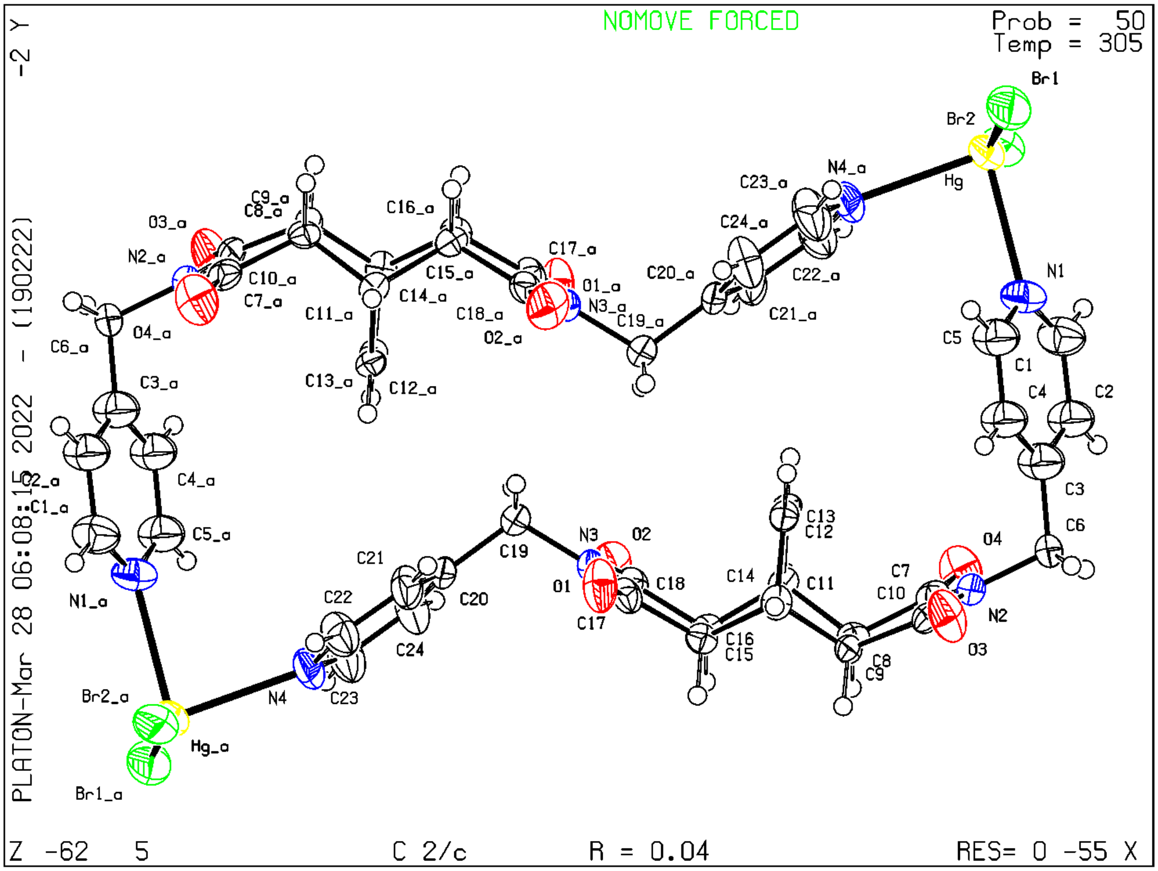

Datablock 6 - ellipsoid plot

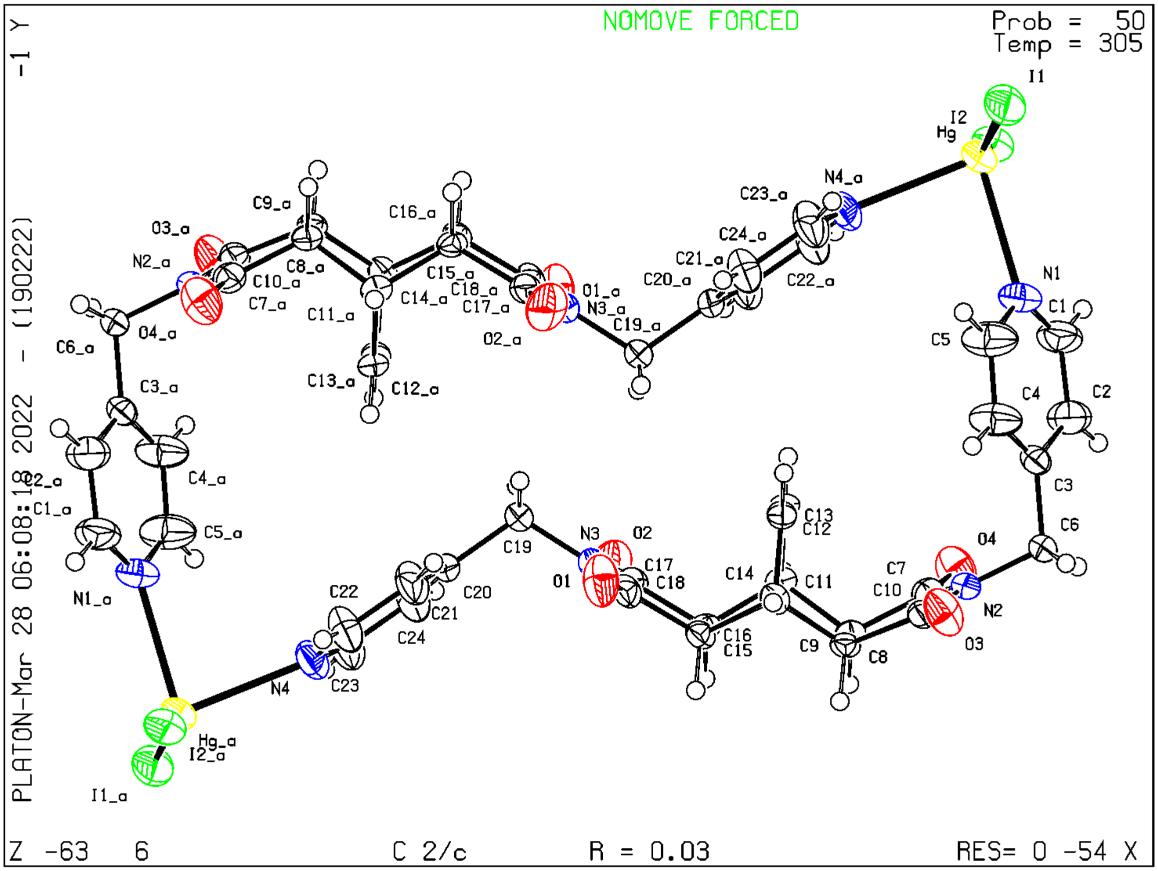

Datablock 7 - ellipsoid plot

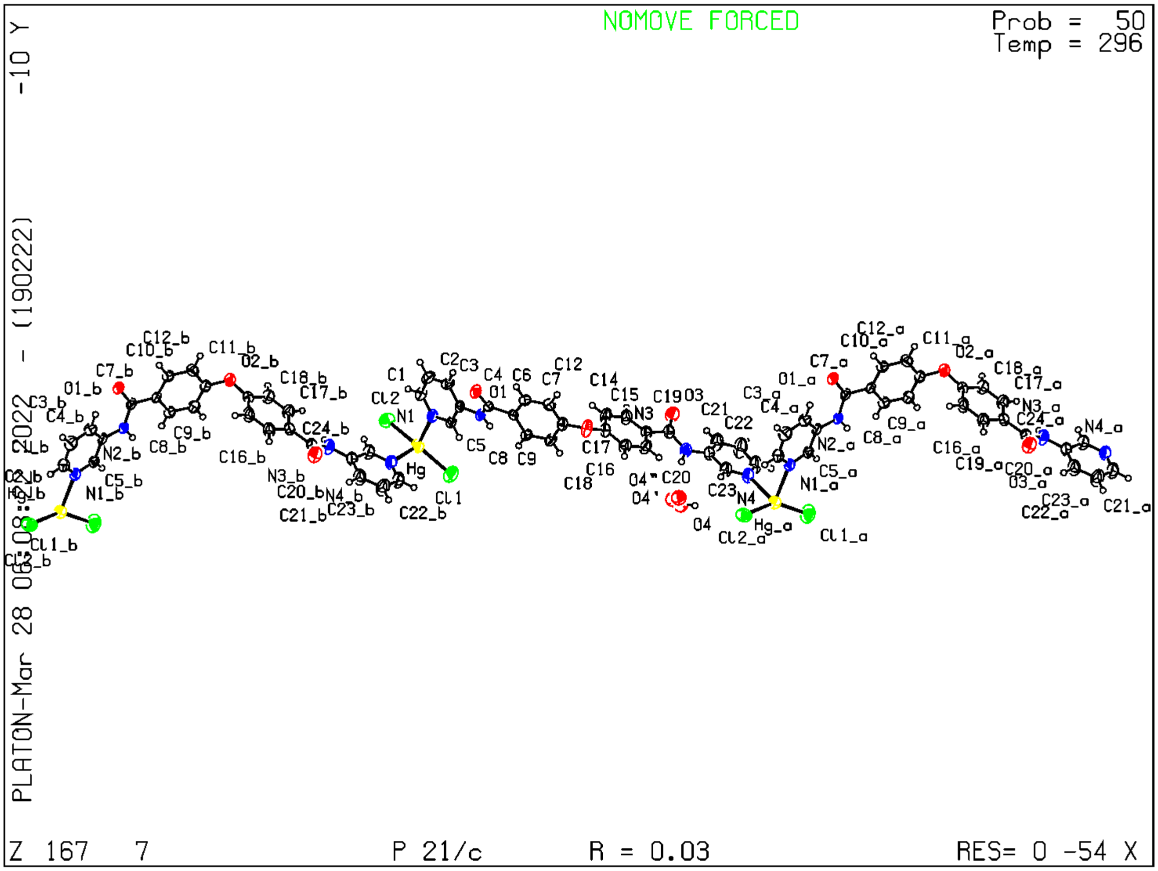

Datablock 8 - ellipsoid plot

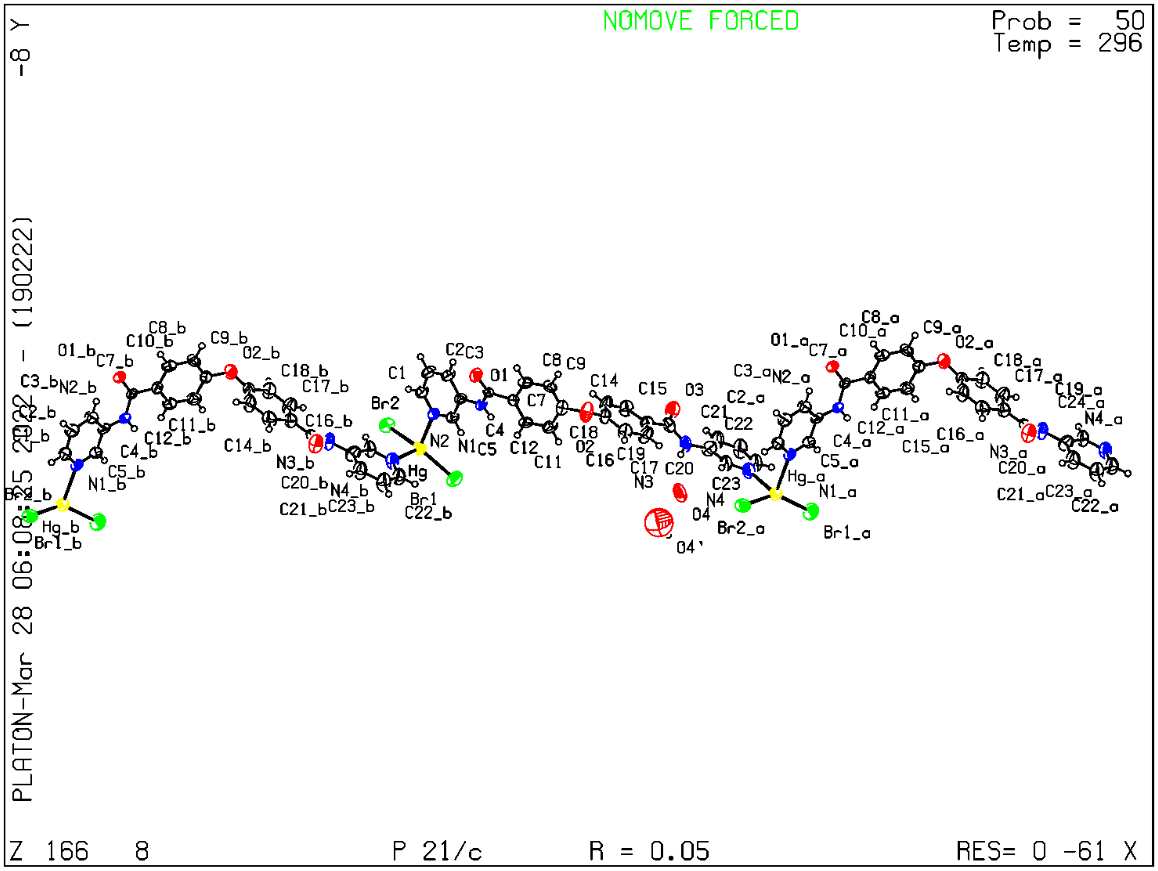

Datablock 9 - ellipsoid plot

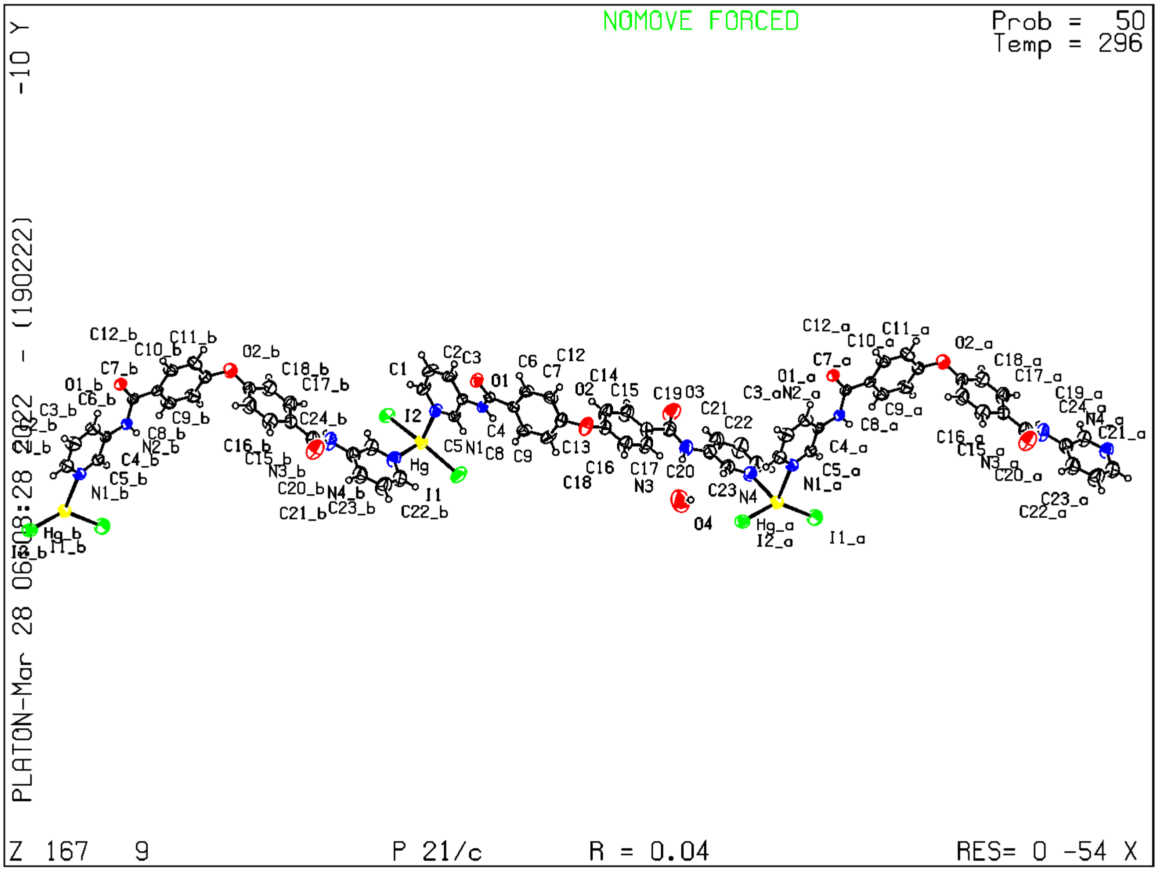

Supplement: Supplementary file 1 [file ijms-23-07861-s001.zip › checkcif.pdf]
