# Supplementary material for: Structural Diversity of Mercury(II) Halide Complexes Containing Bis-pyridyl-bis-amide with Bulky and Angular Backbones: Ligand Effect and Metal Sensing
Source: Int J Mol Sci. 2022 Jul 16;23(14):7861. doi: 10.3390/ijms23147861 (PMC9323401; doi:10.3390/ijms23147861)
Supplement: Supplementary file 1 [file ijms-23-07861-s001.zip › Hg paper supplementry.pdf]

Supplementary Materials

Structural Diversity of Mercury(II) Halide Complexes Containing Bis-pyridyl-bis-amide with Bulky and Angular Backbones: Ligand Effect and Metal Sensing

Manivannan Govindaraj [1], Wei-Chun Huang [1], Chia-Yi Lee [1], Venkatesan Lakshmanan [1], Yu-Hsiang Liu [1], Pamela Berilyn So [2], Chia-Her Lin [2,*] and Jhy-Der Chen [1,*]

[1] Department of Chemistry, Chung-Yuan Christian University, Chung Li, Taoyuan 320, Taiwan; manivannanjent@gmail.com (M.G.); learn122568@gmail.com (W.-C.H.); miss10031031@gmail.com (C.-Y.L.); flower95@gmail.com (V.L.); g10963021@cycu.edu.tw (Y.-H.L.)

[2] Department of Chemistry, National Taiwan Normal University, Taipei 106, Taiwan; pbtiuso@gmail.com

* Correspondence: chiaher@ntnu.edu.tw (C.-H.L.); jdchen@cycu.edu.tw (J.-D.C.); Tel.: +886-3-265-3351 (J.-D.C.)

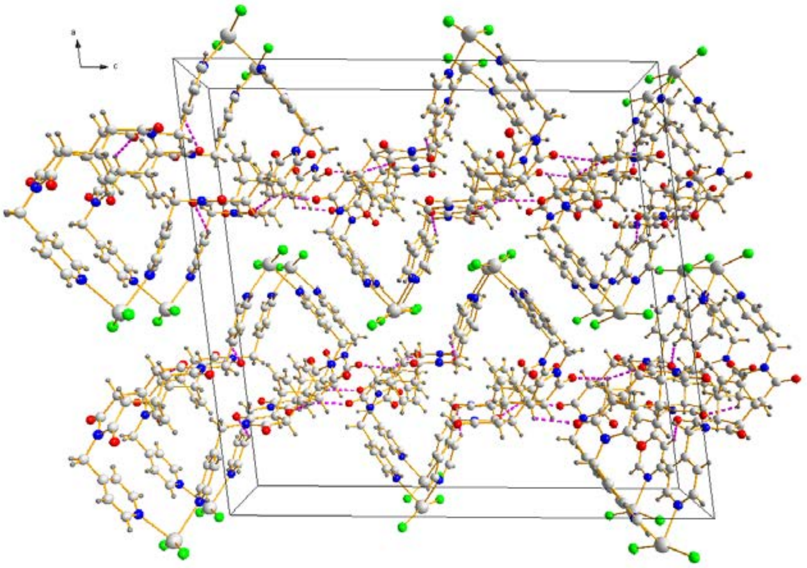

Figure S1. Packing diagram for **4**.

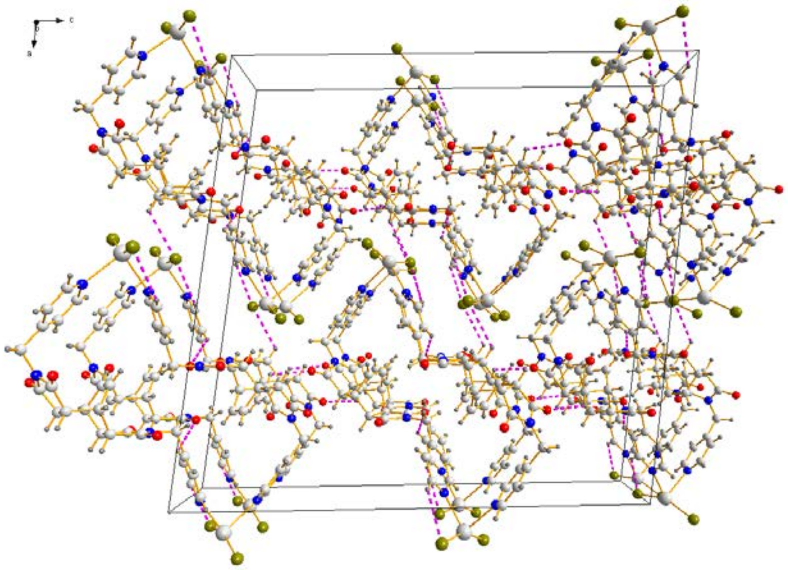

Figure S2. Packing diagram for **5**.

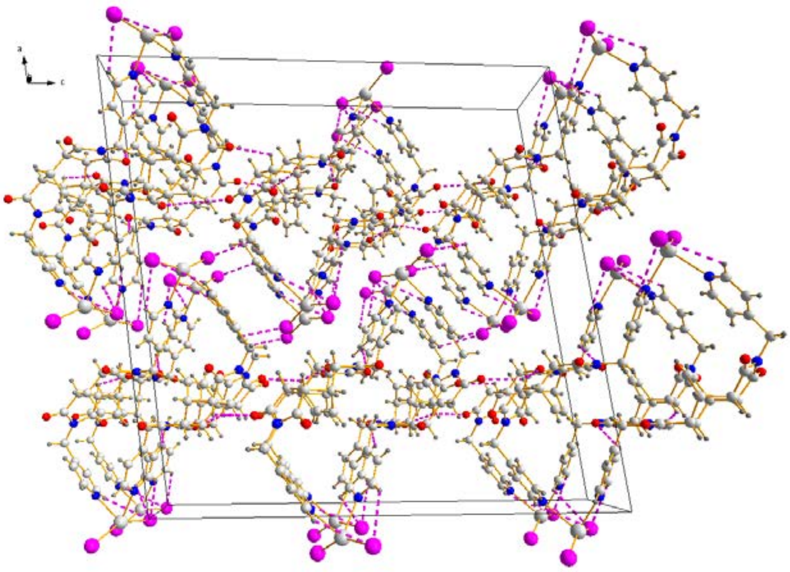

Figure S3. Packing diagram for **6**.

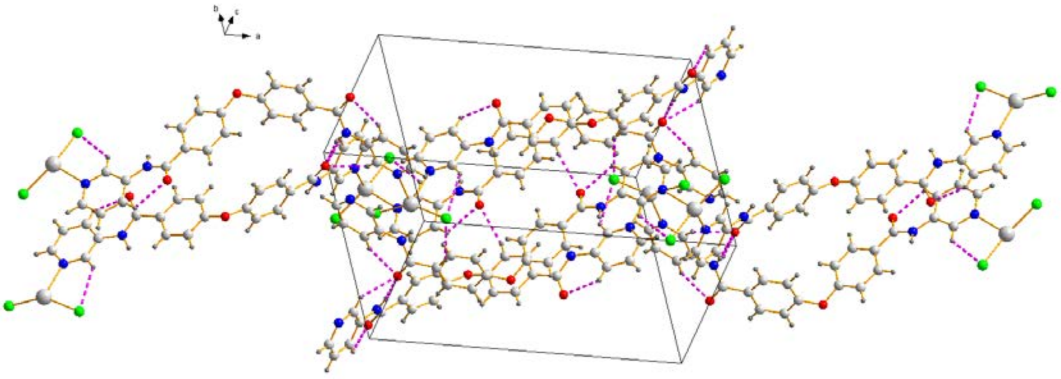

Figure S4. Packing diagram of **7**.

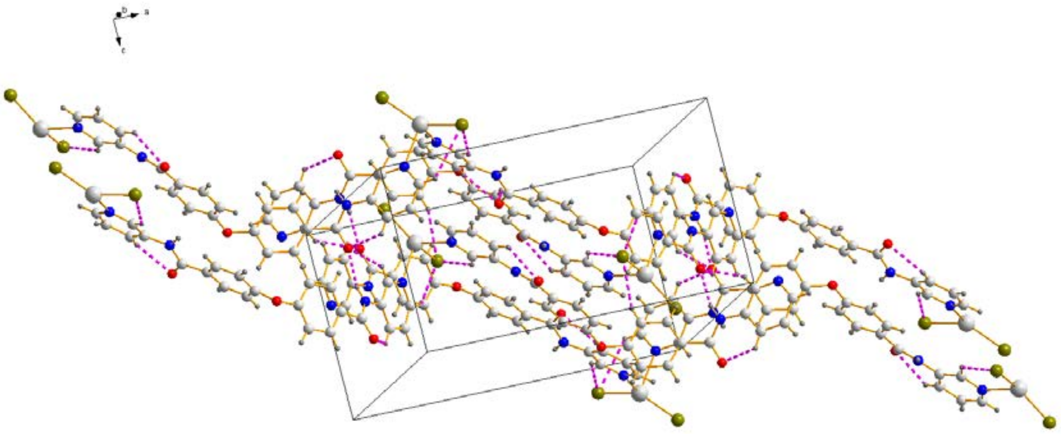

Figure S5. Packing diagram of **8**.

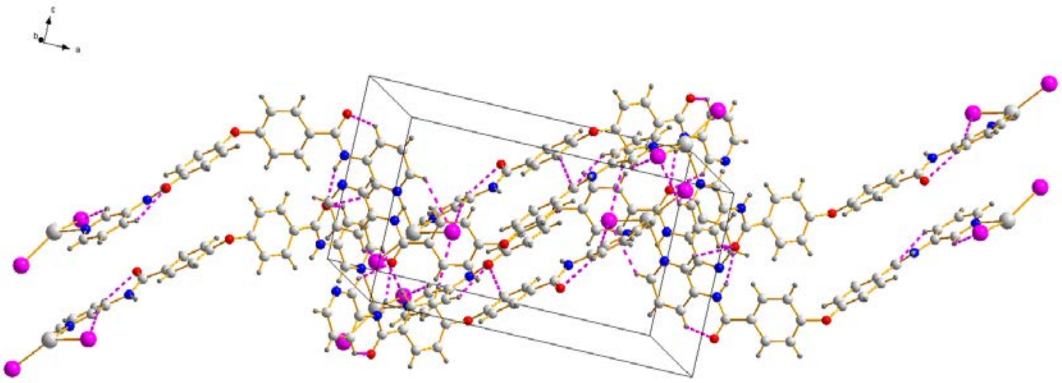

Figure S6. Packing diagram of **9**.

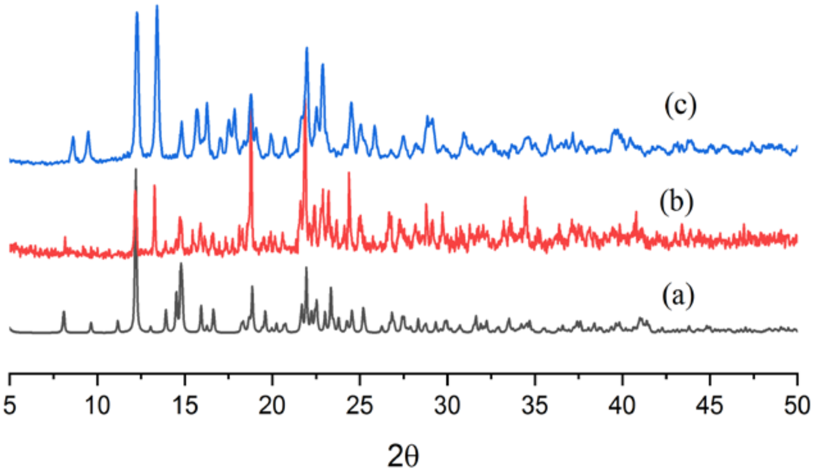

Figure S7. PXRD patterns of **7**. (a) simulated, (b) as synthesized by hydrothermal reaction and (c) as-synthesized by mechanochemical grinding.

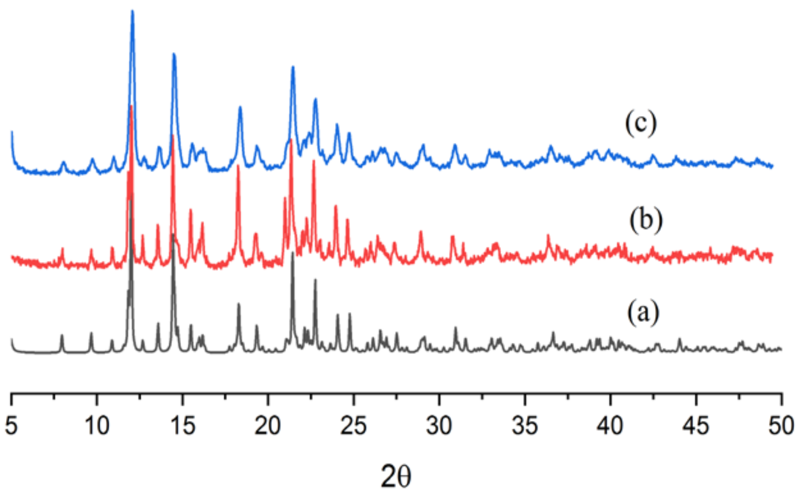

Figure S8. PXRD patterns of **8**. (a) simulated, (b) as synthesized by hydrothermal reaction and (c) as-synthesized by mechanochemical grinding.

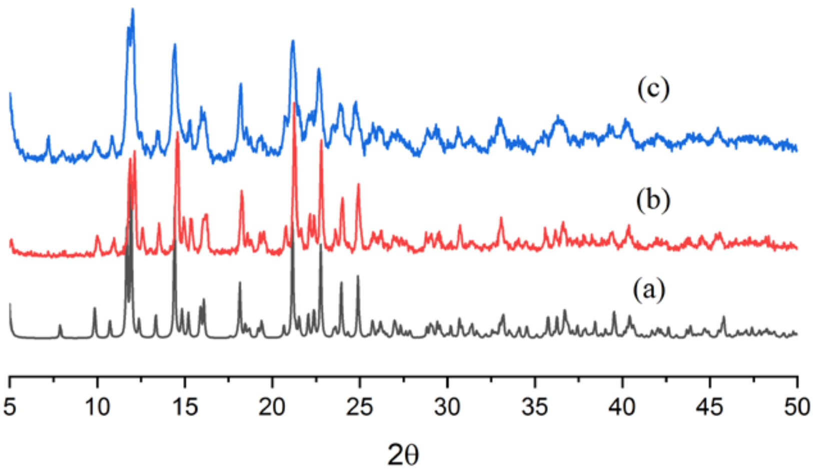

Figure S9. PXRD patterns of **9**. (a) simulated, (b) as synthesized by hydrothermal reaction and (c) as-synthesized by mechanochemical grinding.

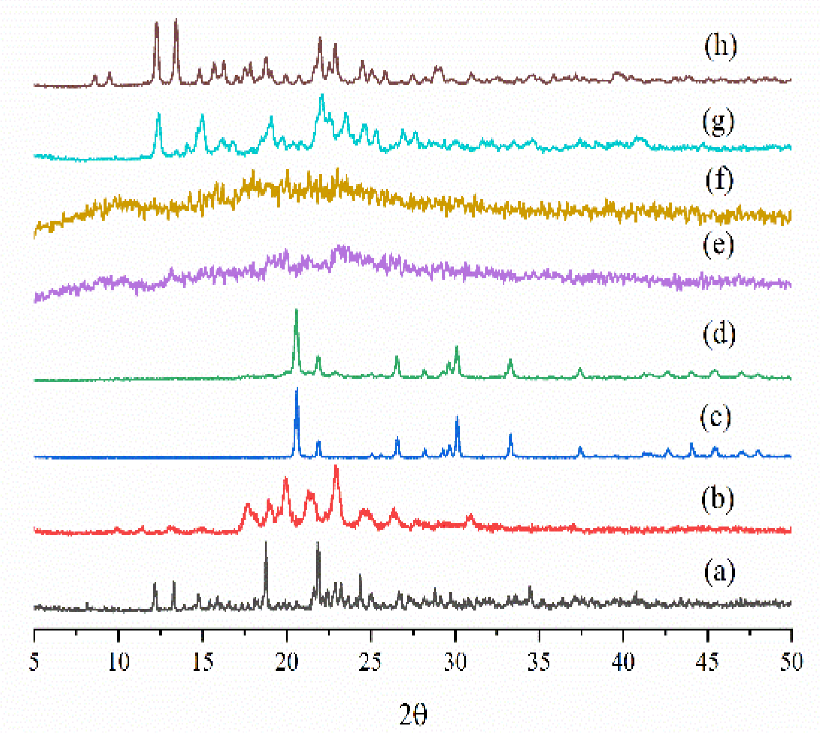

Figure S10. PXRD patterns of (a) as-synthesized of **7**, (b) ligand $\mathbf{L^3}$, (c) $HgCl_2$ and (d) grinding without solvent and grinding with (e) H_2O, (f) DCM, (g) $MeOH/H_2O$ and (h) $EtOH/H_2O$.

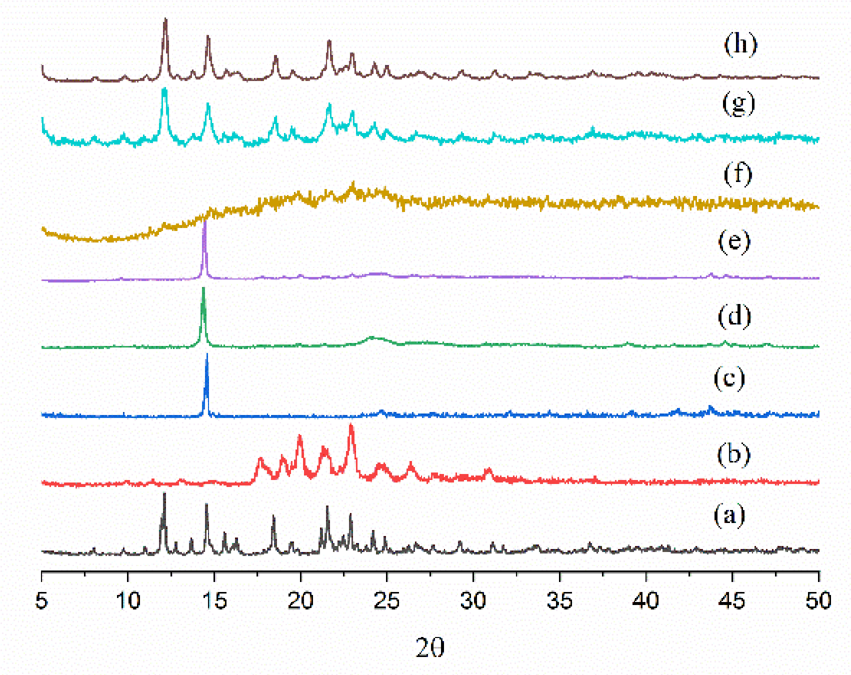

Figure S11. PXRD patterns of (a) as-synthesized of **8**, (b) ligand $\mathbf{L^3}$, (c) $HgBr_2$ and (d) grinding without solvent and grinding with (e) H_2O, (f) DCM, (g) $MeOH/H_2O$ and (h) $EtOH/H_2O$.

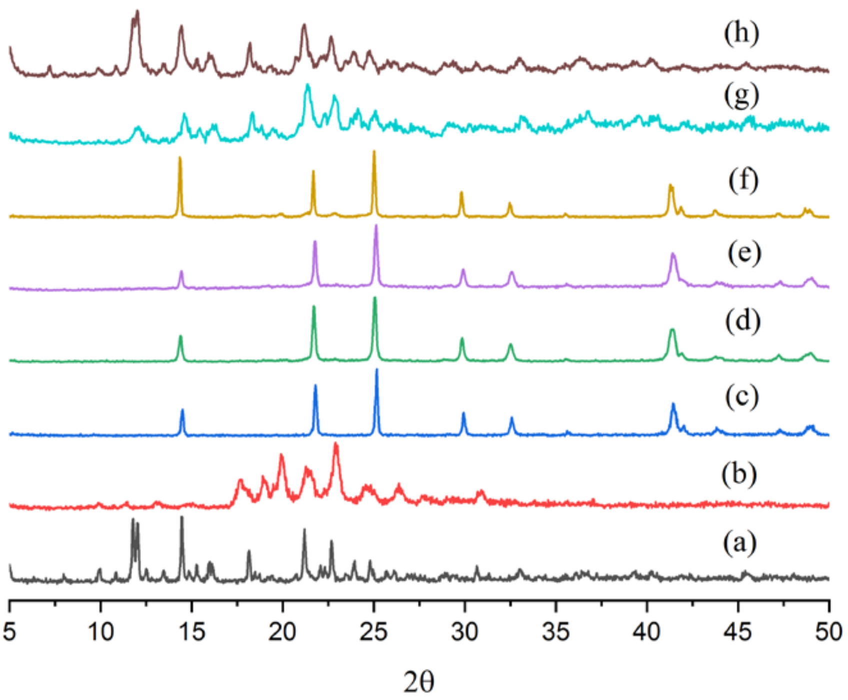

Figure S12. PXRD patterns of (a) as-synthesized of **9**, (b) ligand $\mathbf{L^3}$, (c) HgI_2 and (d) grinding without solvent and grinding with (e) H_2O, (f) DCM, (g) MeOH/H_2O and (h) EtOH/H_2O.

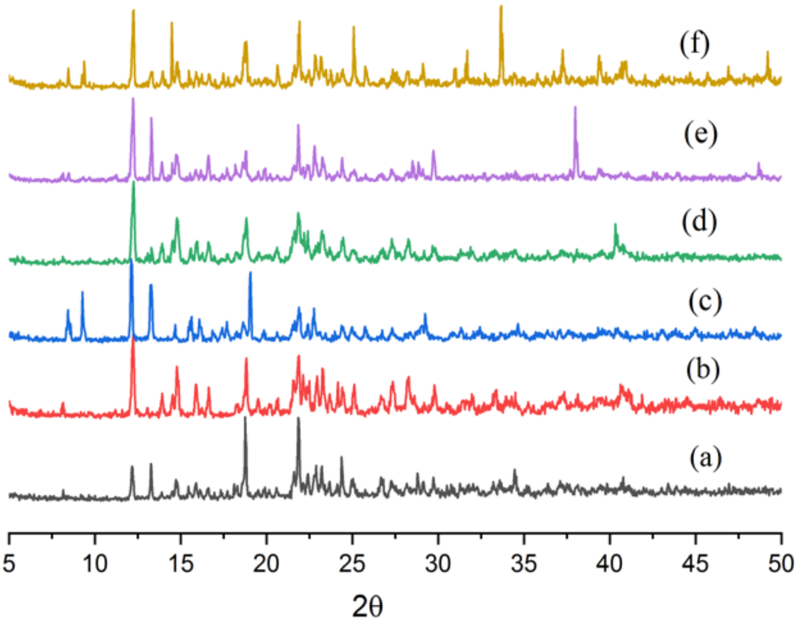

Figure S13. PXRD patterns of **7** in different solvents up to 7 days: (a) as-synthesized, (b) H_2O, (c) EtOH, (d) MeOH, (e) DCM and (f) MeCN.

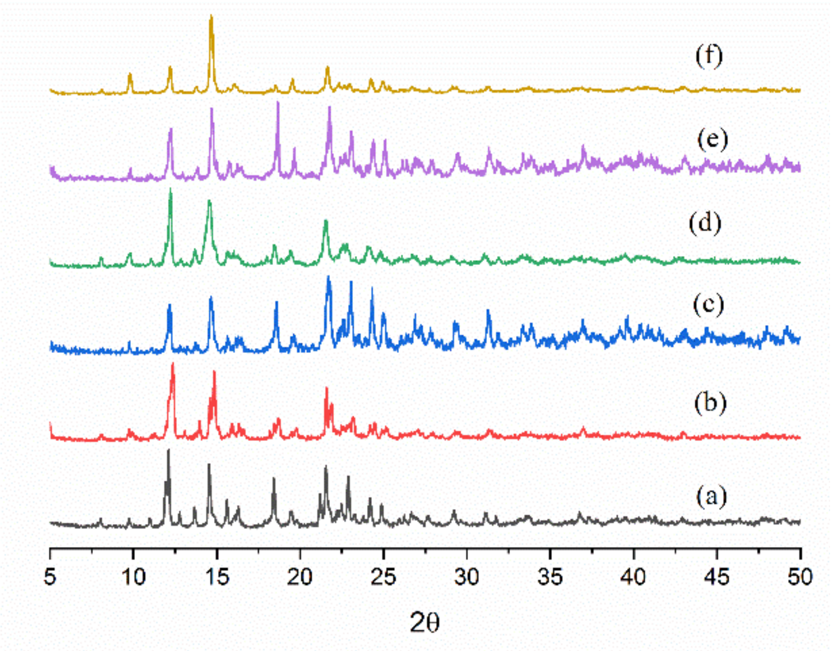

Figure S14. PXRD patterns of **8** in different solvents up to 7 days: (a) as-synthesized, (b) H_2O, (c) EtOH, (d) MeOH, (e) DCM and (f) MeCN.

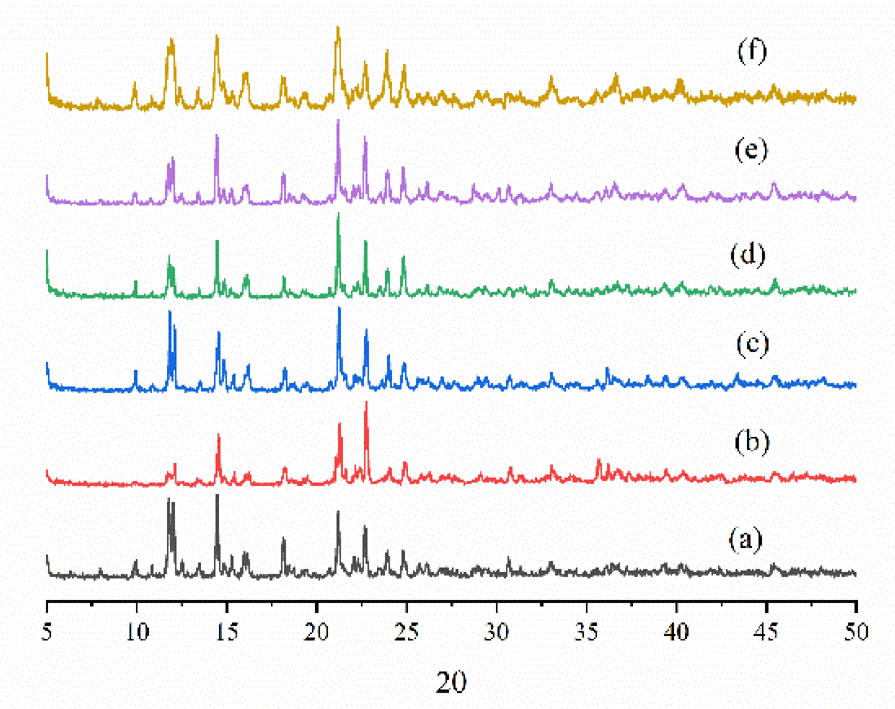

Figure S15. PXRD patterns of **9** in different solvents up to 7 days: (a) as-synthesized, (b) H_2O, (c) EtOH, (d) MeOH, (e) DCM and (f) MeCN.

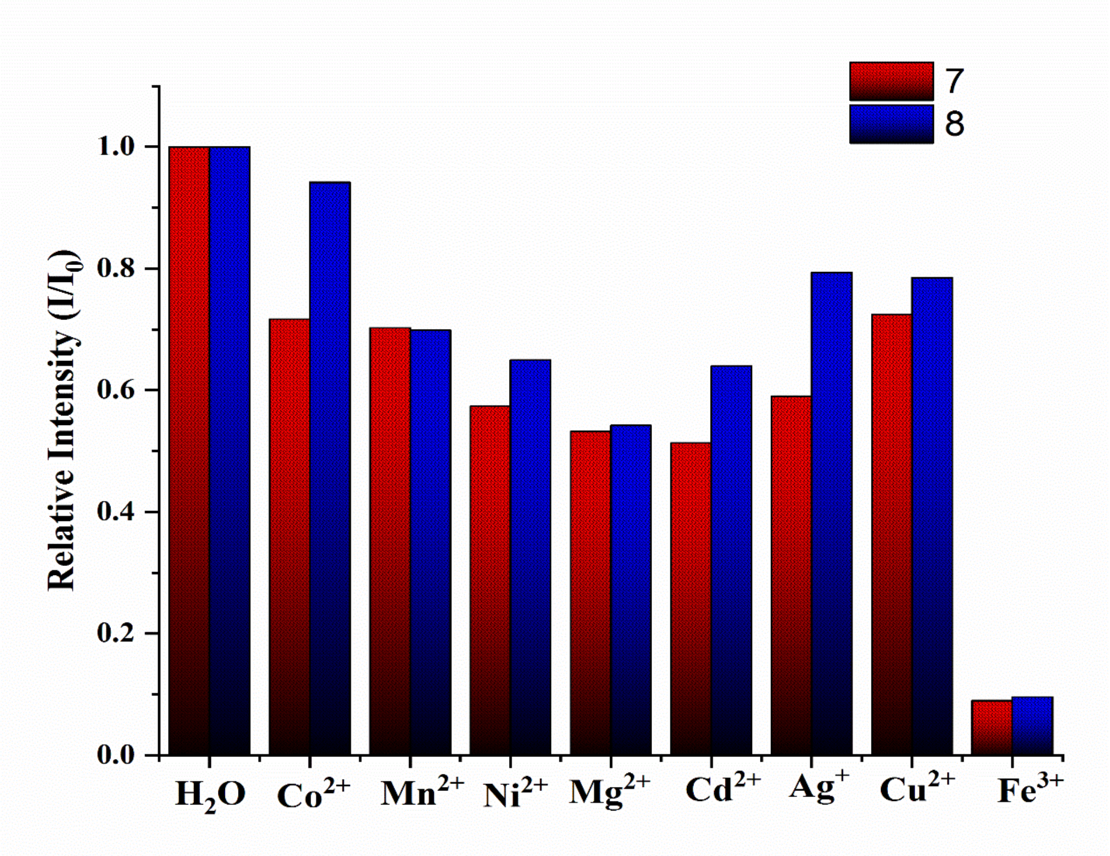

Figure S16. A diagram showing the dependence of the emission intensities of complexes **7** and **8** in various metal ions.

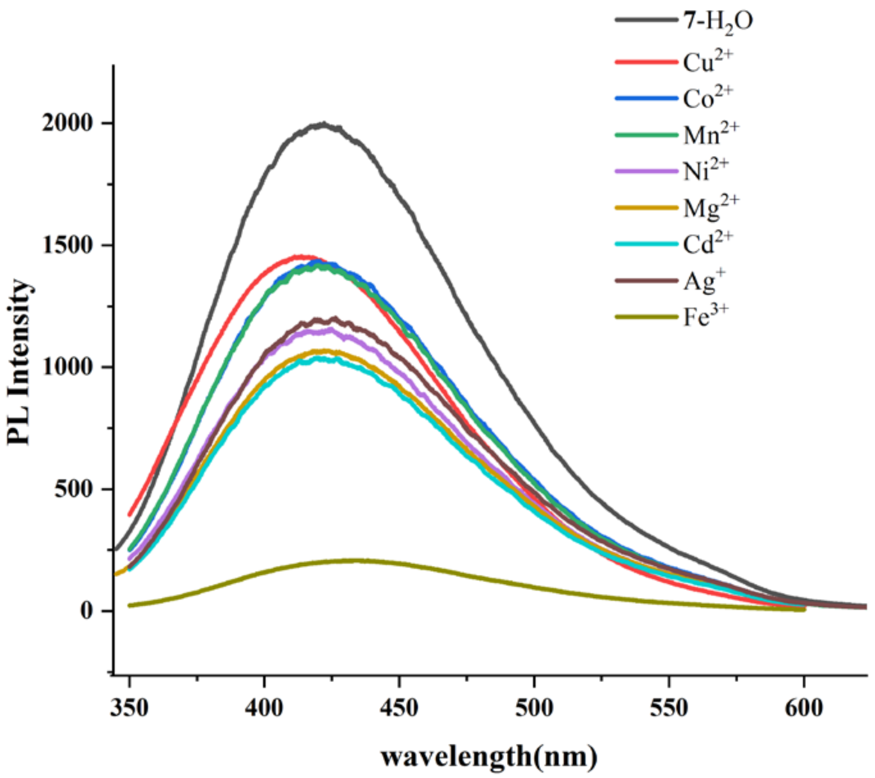

Figure S17. Solid-state emission spectra of **7** after immersion into different metal ions.

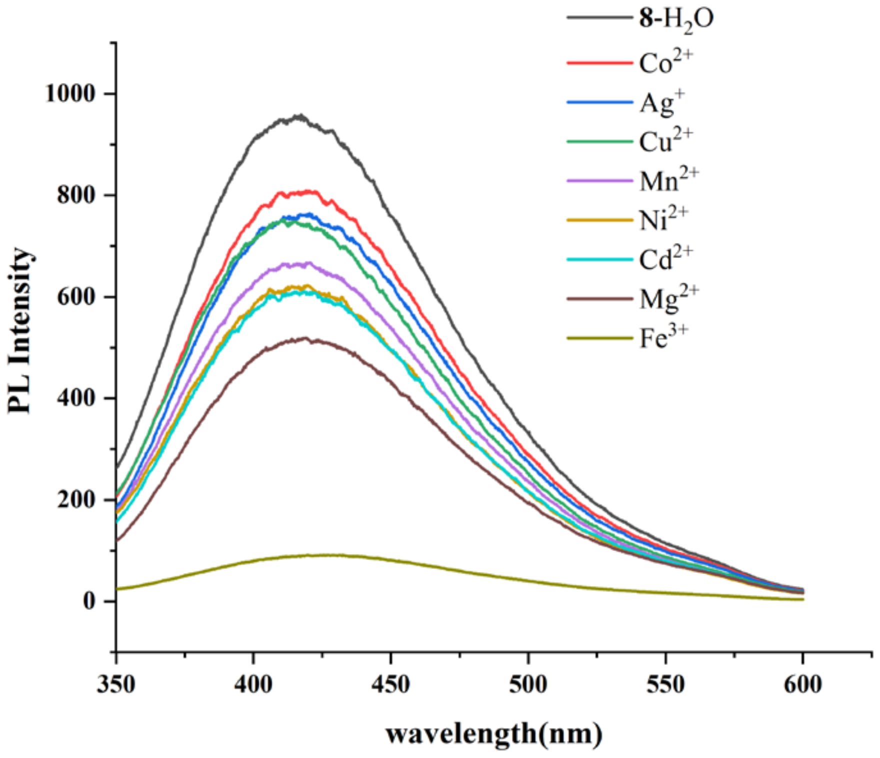

Figure S18. Solid-state emission spectra of **8** after immersion into different metal ions.

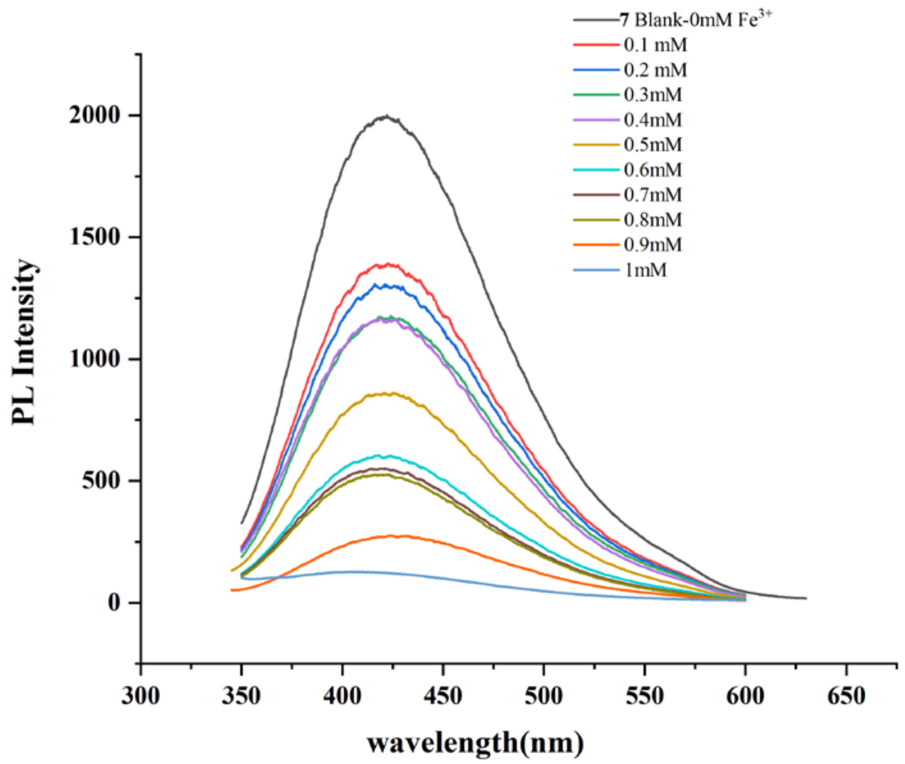

Figure S19. Dependence of the emission intensities of **7** in Fe^{3+} with various concentrations.

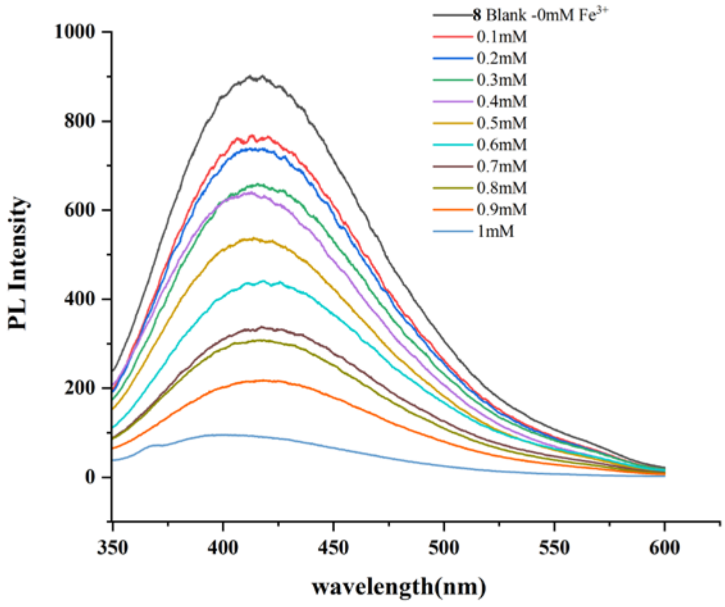

Figure S20. Dependence of the emission intensities of **8** in Fe^{3+} with various concentrations.

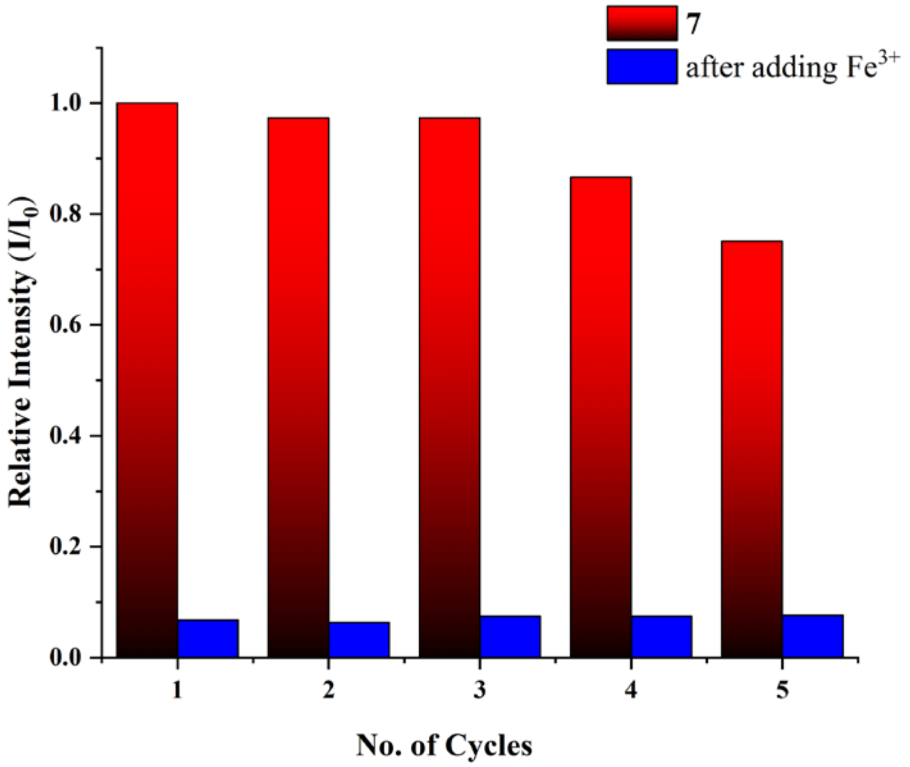

Figure S21. Bar diagrams showing the emission intensities (λ_{ex} = 325 nm) of **7** treated with Fe^{3+} for five repeated cycles.

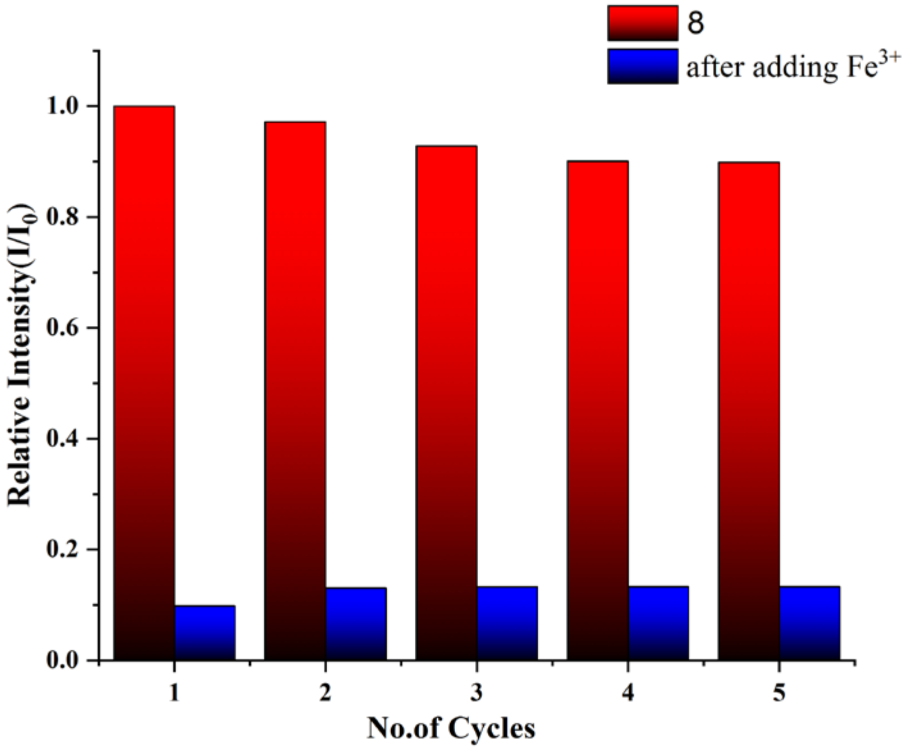

Figure S22. Bar diagrams showing the emission intensities (λ_{ex} = 326 nm) of **8** treated with Fe^{3+} for five repeated cycles.

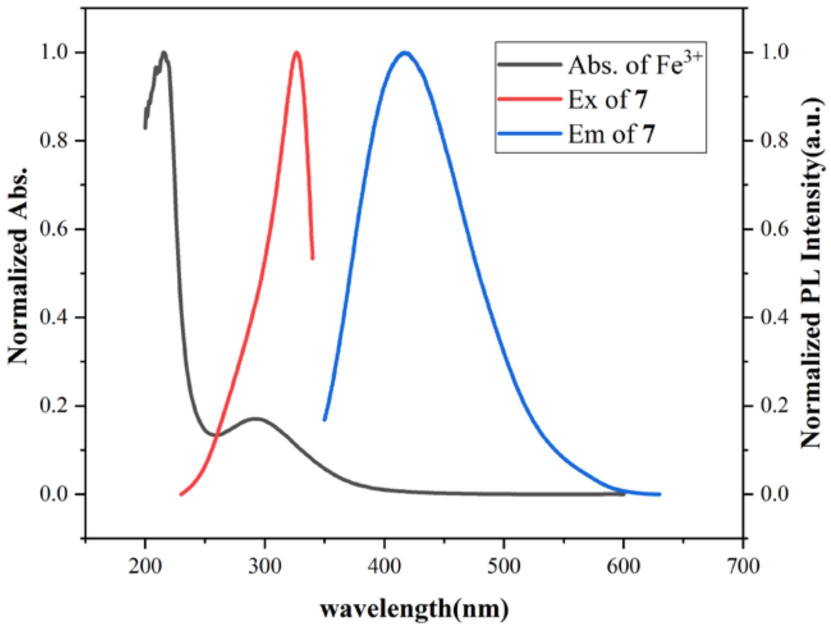

Figure S23. UV-vis absorption spectrum of Fe^{3+} ions in aqueous solution and the excitation and emission spectra of **7**.

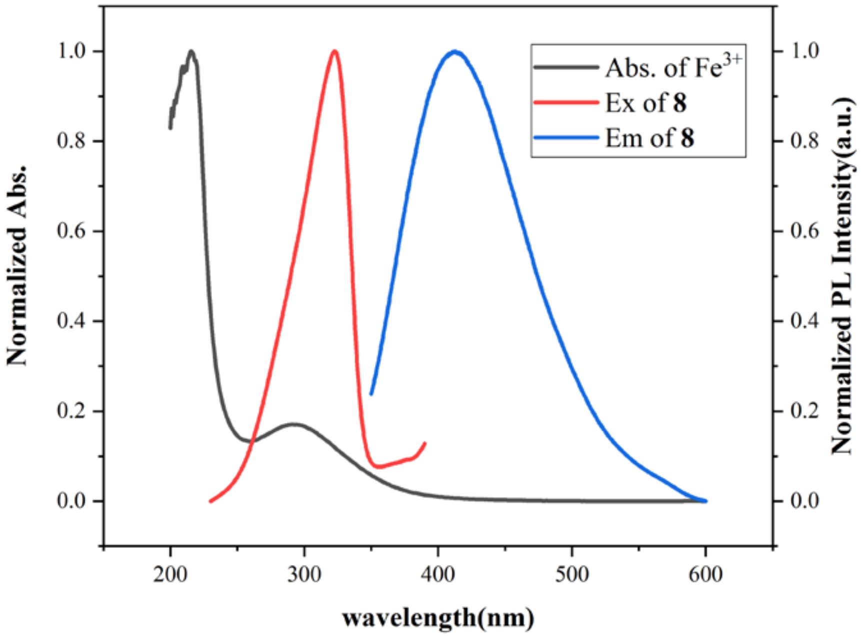

Figure S24. UV-vis absorption spectrum of Fe^{3+} ions in aqueous solution and the excitation and emission spectra of **8**.

Element	Weight%	Atomic%
C K	45.08	63.20
N K	10.18	12.24
O K	17.49	18.41
Cl K	8.62	4.09
Fe L	2.26	0.68
Hg M	16.37	1.37
Totals	100.00	

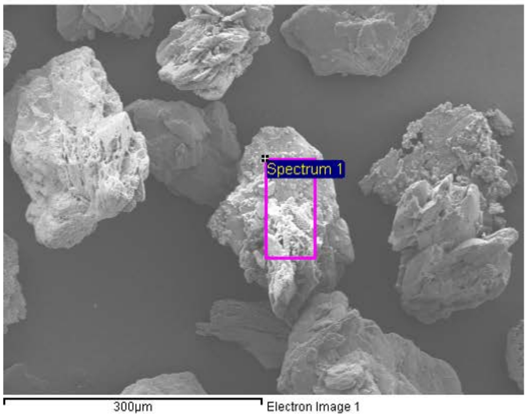

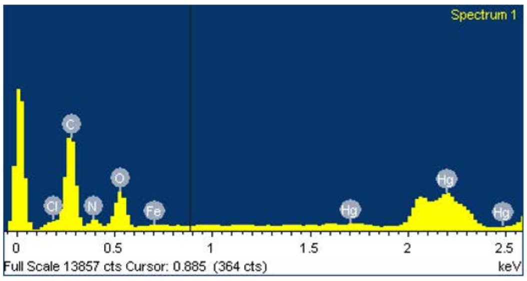

Figure S25. EDX data for Fe^{3+}-uptaked complex **7**.

Element	Weight%	Atomic%
C K	39.57	66.52
N K	7.06	10.17
O K	10.93	13.79
Fe L	4.57	1.65
Br L	26.64	6.73
Hg M	11.25	1.13
Totals	100.00	

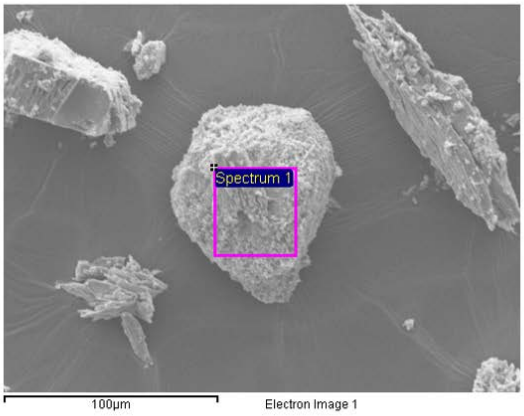

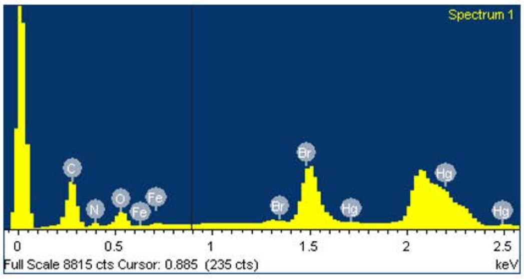

Figure S26. EDX data for Fe^{3+}-uptaked complex **8**.

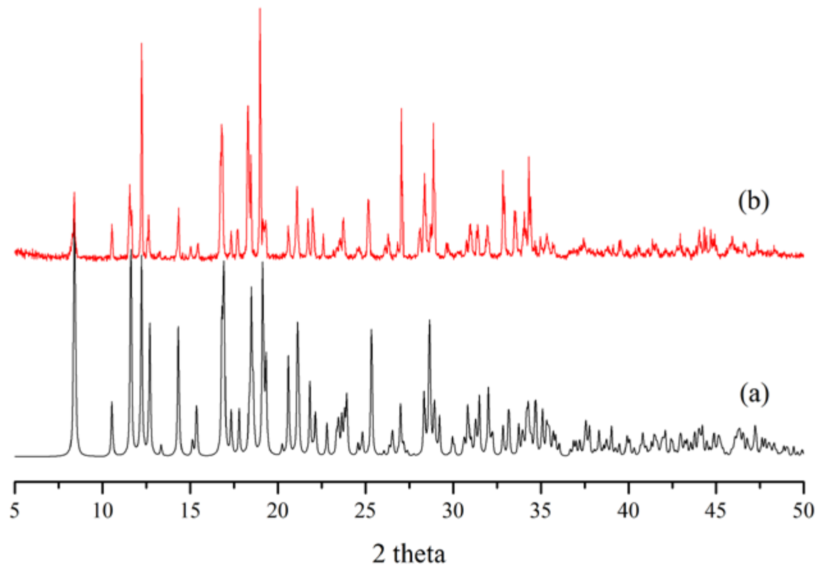

Figure S27. PXRD patterns of complex **1**. (a) simulated, (b) as synthesized by hydrothermal reaction.

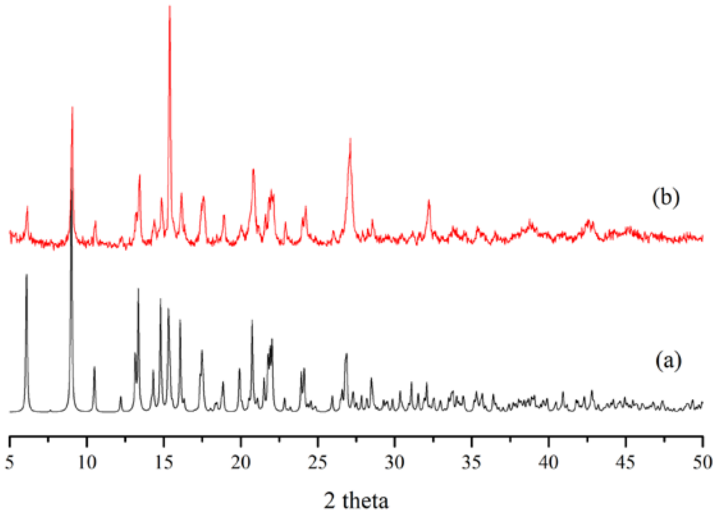

Figure S28. PXRD patterns of complex **2**. (a) simulated, (b) as synthesized by hydrothermal reaction.

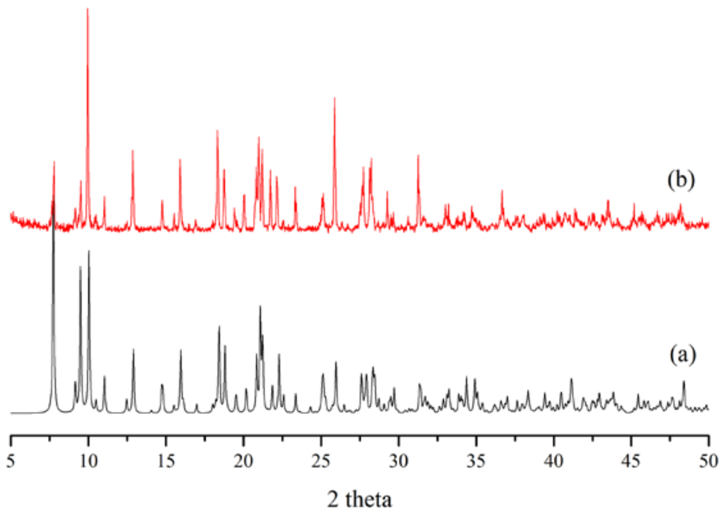

Figure S29. PXRD patterns of complex **3**. (a) simulated, (b) as synthesized by hydrothermal reaction.

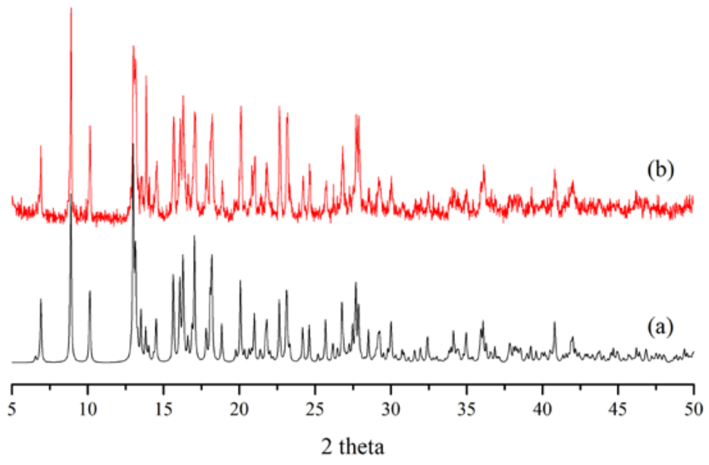

Figure S30. PXRD patterns of complex **4**. (a) simulated, (b) as synthesized by hydrothermal reaction.

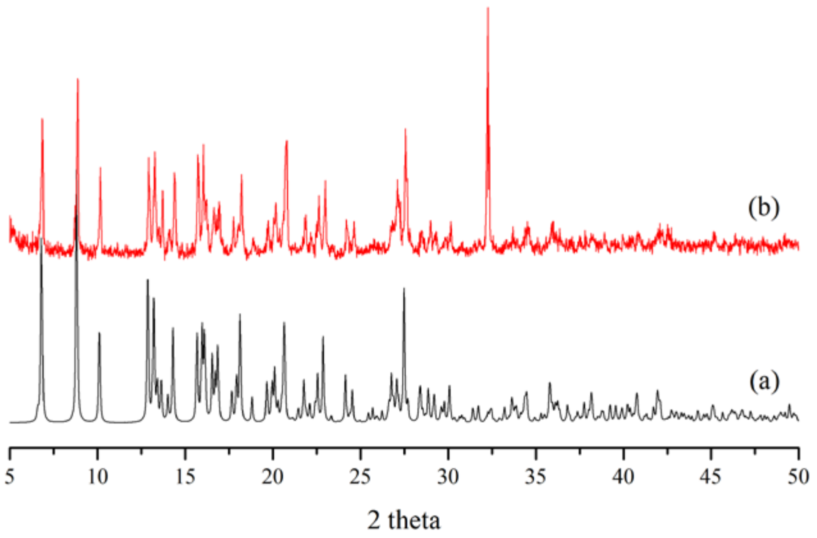

Figure S31. PXRD patterns of complex **5**. (a) simulated, (b) as synthesized by hydrothermal reaction.

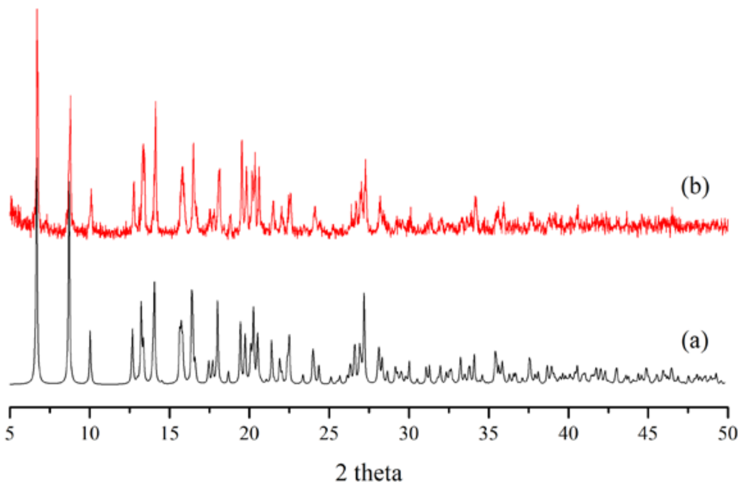

Figure S32. PXRD patterns of complex **6**. (a) simulated, (b) as synthesized by hydrothermal reaction.
